# Froth Flotation of Chalcopyrite/Pyrite Ore: A Critical Review

**DOI:** 10.3390/ma15196536

**Published:** 2022-09-21

**Authors:** César I. Castellón, Norman Toro, Edelmira Gálvez, Pedro Robles, Williams H. Leiva, Ricardo I. Jeldres

**Affiliations:** 1 Departamento de Ingeniería Química y Procesos de Minerales, Facultad de Ingeniería, Universidad de Antofagasta, Angamos Avenue 601, Antofagasta 1240000, Chile; 2 Faculty of Engineering and Architecture, Universidad Arturo Prat, Almirante Juan José Latorre 2901, Antofagasta 1244260, Chile; 3 Department of Metallurgical and Mining Engineering, North Catholic University, Angamos Avenue 0610, Antofagasta 1270709, Chile; 4 Escuela de Ingeniería Química, Pontificia Universidad Católica de Valparaíso, Valparaíso 2340000, Chile

**Keywords:** chalcopyrite flotation, pyrite depressants, mineral oxidation, collector adsorption, pH modifiers, modern collectors

## Abstract

In the present work an intense bibliographic search is developed, with updated information on the microscopic fundamentals that govern the behavior of flotation operations of chalcopyrite, the main copper mineral in nature. In particular, the effect caused by the presence of pyrite, a non-valuable mineral, but challenging for the operation due to its ability to capture a portion of collector and float, decreasing the quality of the concentrate, is addressed. This manuscript discusses the main chemical and physical mechanisms involved in the phenomena of reagent adsorption on the mineral surface, the impact of pH and type of alkalizing agent, and the effect of pyrite depressants, some already used in the industry and others under investigation. Modern collector reagents are also described, for which, although not yet implemented on an industrial scale, promising results have been obtained in the laboratory, including better copper recovery and selectivity, and even some green reagents present biodegradable properties that generate a better environmental perspective for mineral processing.

## 1. Properties and Structure of Chalcopyrite

Natural chalcopyrite has a molecular weight of 183.5 g/mol, with a theoretical composition of 34.6% Cu, 30.4% Fe, and 34.9% S. This mineral has a brassy yellow color, often with an iridescent tarnish, including a bronze color. Sometimes it occurs with a surface layer that gives various bright shades due to oxidation. The oxidation occurs through the initial formation of ferrous (FeS_2_O_3_) or cuprous (Cu_2_S_2_O_3_) thiosulfates, which are further oxidized to give ferric or cupric sulfates [1]. It has a greenish-black streak with a Mohs hardness of between 3½ and 4. Its density is approximately 4.2 g/cm^3^ with a melting point of roughly 880 °C.

**Figure 1:**
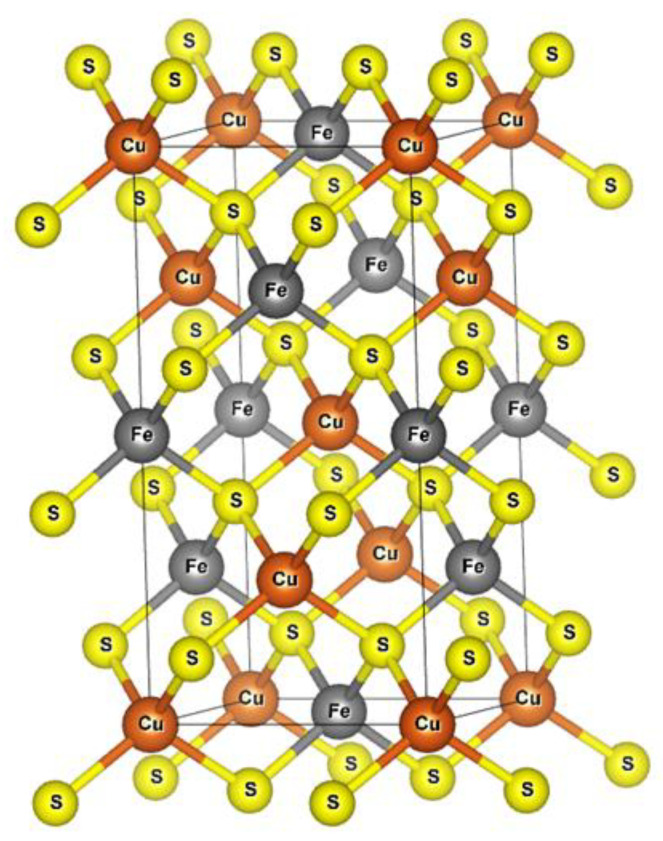
Unit cell model of chalcopyrite.

The typical crystal structure is a simple tetragonal organization (α-chalcopyrite) with cell dimensions of a = b = 5.289 Å and c = 10.423 Å, with a bond length of Cu–S = 2.302 Å and Fe-S = 2.2566 Å [2]. The structure is related to the unit cell of sphalerite (ZnS), where the four zinc atoms are replaced by two copper atoms and two iron atoms [3] located at the tetrahedron angles and with a specific order in each plane [4] (Figure 1). However, two other structures have also been found in the literature, i.e., the cubic form with the molecular formula of CuFeS_1.82_, known as β-chalcopyrite, and γ-chalcopyrite with the molecular formula of CuFeS_2−_x, where x can take on a wide range of values.

Nowadays, froth flotation of chalcopyrite ore has developed a large number of reagents that make it possible to improve the mineral’s buoyancy conditions, both in the presence of ions that simulate the use of seawater or process water, and in conjunction with other minerals. However, despite all the advances made in this field, the flotation process still suffers from drawbacks such as low efficiency in the recovery of chalcopyrite, as does the chemistry involved in the process. Therefore, the aim of this review is to outline the scientific achievements in the field of froth flotation of chalcopyrite and its separation from other minerals, as well as to evaluate and review new advances in the chalcopyrite flotation process, while understanding the basic theory. The focus is on chalcopyrite–pyrite separation. The effects of clays with seawater have not been discussed in depth, considering that there are two new reports critically reviewing this subject, which are referred to in the text [5,6].

## 2. Chalcopyrite Mineral Oxidation

The oxidation of the mineral directly influences the performance of the flotation, which is developed mainly with the support of collectors, although it can eventually be carried out without the use of chemical reagents. Understanding this phenomenon helps in optimizing the operation, which includes the oxidation rate, surface processes, and generated products. The factors that most influence the transfer of electrons from the more anodic to the more cathodic material are the pulp potential [5], temperature [6,7], pH [8], and the interactions between the minerals themselves during grinding (galvanic interaction) or conditioning.

**Figure 2:**
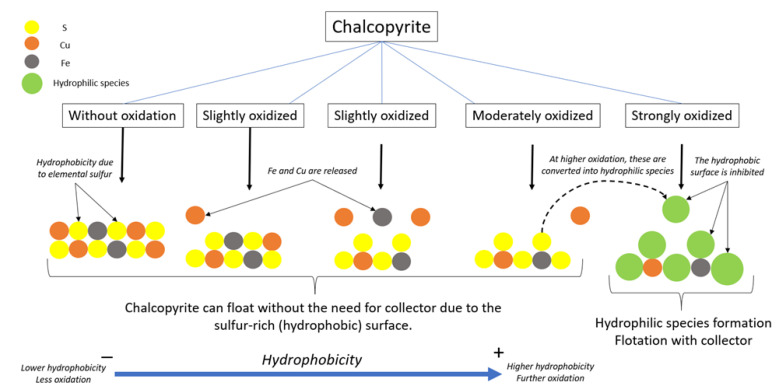
Sulfide oxidation mechanisms (adapted from Aghazadeh et al. [7]).

Chalcopyrite can be floated without a collector once elemental sulfide forms on its surface, a characteristic that is acquired after surface oxidation [9]. It has been suggested that elemental sulfur and metal-deficient sulfides (M_1−x_S) are the hydrophobic species that may appear on the surface, giving the mineral a greater floatability. Then, flotation can occur if metal oxides and hydroxides are solubilized in the medium. However, excessive oxidation can form thiosalts and sulfates, which react with previously dissolved metal ions and re-adsorb them to the surface, creating a hydrophilic layer that impairs the process (Figure 2).

**Figure 3:**
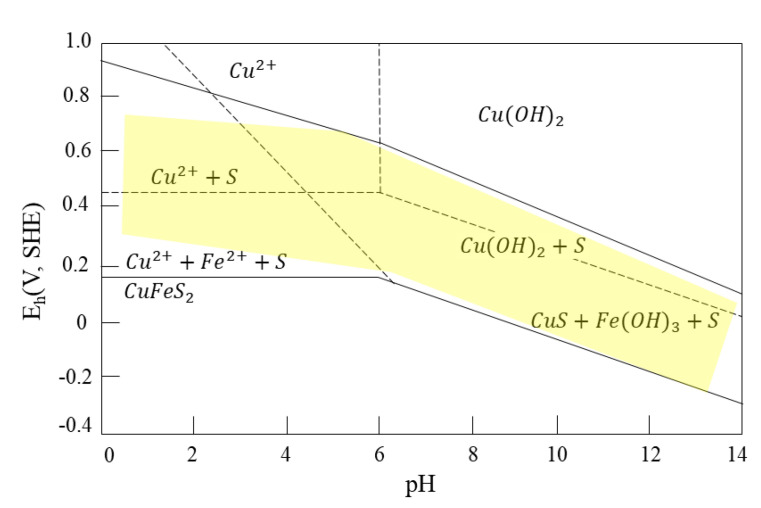
Eh-pH diagram for chalcopyrite with elemental sulfur as metastable phase (adapted from Hu et al. [8]).

The Eh–pH diagram (Figure 3) shows the area where chalcopyrite exhibits self-induced floatability, which is precisely the region with metastable elemental sulfur (a hydrophobic species). When the conditions are above the upper limit, hydrophilic species such as thiosulfate and metal hydroxides form, and flotation ceases. Based on the Pourbaix diagram (Figure 3), it is possible to make a prediction of the mineral’s floatability. This figure also shows that in a broad range of pH and potential, chalcopyrite exhibits the best self-induced floatability, and it has wide lower and upper limits of potential that induce its natural ability to float naturally [8].

It has also been reported that sulfide minerals are semiconductors that can accept or donate electrons in an electrochemically active system [9,10,11,12] and their level of floatability is closely related to the pulp potential and the existence of thiolate or dithiolate radicals [13]. This is because some minerals need the thiolate radicals to float, while others need the dithiolate radicals. This depends on whether they are p-type (positively charged) or n-type (negatively charged) semiconductors. The p-type mineral semiconductors require a negatively charged collector radical to be adsorbed on their surface to become hydrophobic. In the case of n-type semiconductors with a negatively charged surface, the thiolate radical will not be able to adsorb on its surface. Instead, the weaker dithiolate will form a type of hydrogen bond on this surface, allowing the mineral to become hydrophobic [12].

At low pH (approximately pH 5), the iron dissolves from the mineral, forming a hydrophobic surface deficient in iron and rich in sulfur, thus resulting in a higher flotation rate (Equation (1)). At high pH (pH > 9), the iron form a hydroxide on the surface, which, being a hydrophilic species, decreases the flotation rate (Equation (2)).

(1) $$MS+\frac{1}{2}nO_{2}+2nH^{+}\overset{\;}{↔}M_{1-n}S+nM^{2+}+nH_{2}O\;$$

(2) $$MS+\frac{1}{2}nO_{2}+nH_{2}O\overset{\;}{↔}M_{1-n}S+nM{\left(OH\right)}_{2}\;$$

**Figure 4:**
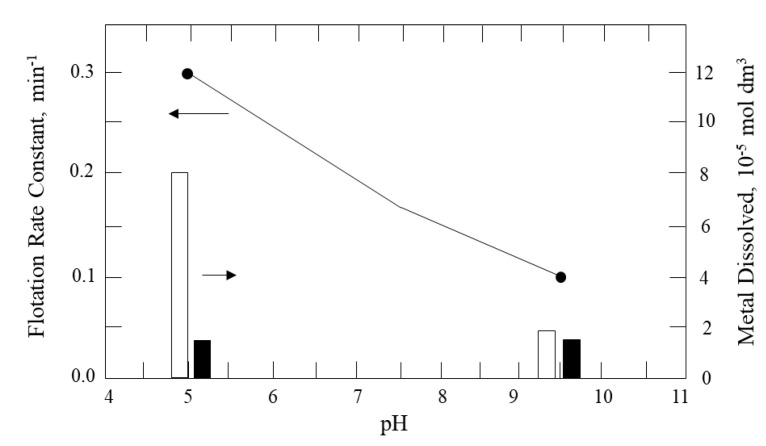
Flotation rate constant of chalcopyrite without collector as a function of pH. The dissolution of iron and copper is represented by the white and black bars, respectively (conditioning with N_2_ gas) (adapted from Fairthorne et al. [14]).

Fairthorne et al. [14] observed that the dissolution of copper under acidic pH was much lower than that of iron, while at pH > 9, the dissolution of both species was comparable. Figure 4 shows the flotation kinetic constant of chalcopyrite without a collector and the concentration of metal dissolved in solution, considering water prepared with KCl 0.01 mol/L and modifying the pH with concentrated solutions of hydrochloric acid or potassium hydroxide. The parameters in the figure were obtained by fitting the Garcia-Zuniga first-order flotation kinetic model [15].

The accepted mechanism for the initial stage of the sulfide mineral oxidation involves the migration of the metal from the outermost layers to the external surface, followed by dissolution in acid solutions or formation of a metal hydroxide layer in an alkaline environment. In an acidic or neutral environment, the oxidation of chalcopyrite produces soluble cations and sulfate. In contrast, in an alkaline environment, polythionates, sulfate, and thiosulfate are produced, which results in decreased recovery of chalcopyrite [16].

The formation of a hydrophobic layer deficient in iron metal or elemental sulfur on the surface allows floatability of the chalcopyrite; however, this is affected by the interaction with the several mineralogical species present in the pulp or by adsorption of precipitates (e.g., iron hydroxides) [17]. Even the presence of Pb (II) or Zn (II) ions at low concentrations (<100 ppm) can influence chalcopyrite recovery. In most industrial operations, the use of a collector is necessary to ensure its hydrophobic character or provide induced hydrophobicity [18].

## 3. Chalcopyrite Flotation Collectors

When a particle collides with the bubble, the possibility of attaching depends fundamentally on the hydrophobic nature of the mineral surface. Although minerals present natural floatability, this condition is often insufficient to achieve industrially sustainable performance. Therefore, it is common to use chemical reagents, called collectors, which seek the surface of the valuable minerals (ideally selectively) and modify their interfacial properties.

**Figure 5:**
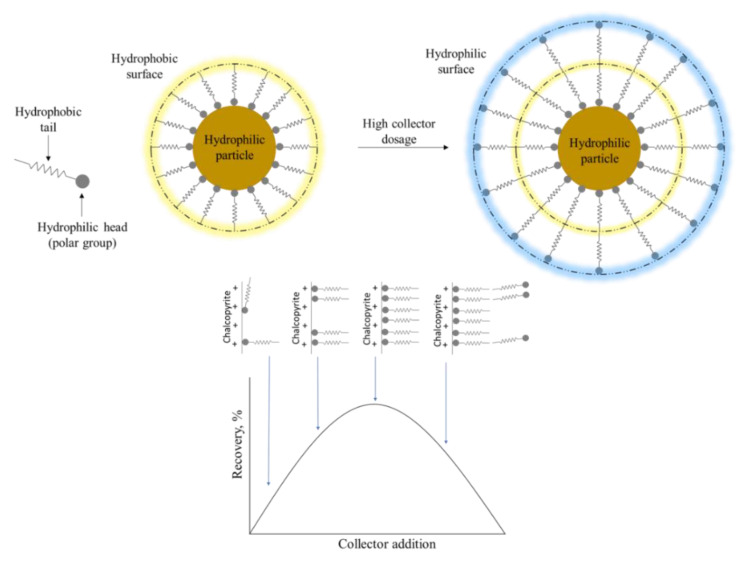
Schematic of the hydrophobic coating and how it affects the relationship between collector concentration and recovery.

In order for the collectors to adhere to the surface and impart hydrophobicity, they must have two functionalities in their molecule: a hydrophilic group that is capable of chemically or electrostatically interacting on the surfaces of mineral species and a non-polar group composed of an aliphatic chain that extends towards the liquid phase, providing the hydrophobic character. An appropriate dose can provide maximum performance, but excess may lead to low recoveries due to double-layer adsorption [19,20]. Once the collector molecules cover the entire surface, the hydrocarbon chains of the first layer with excess collector molecules will interact by hydrophobic attraction, exposing the polar part to the solution (see Figure 5). The collector is first attached to the mineral surface, where adsorption occurs easily if the ore is not oxidized. As the dose increases, the molecules bind and allow the hydrophobic interactions of their tails to stabilize the adsorbed collector.

### 3.1. Chalcopyrite–Pyrite Selectivity

**Figure 6:**
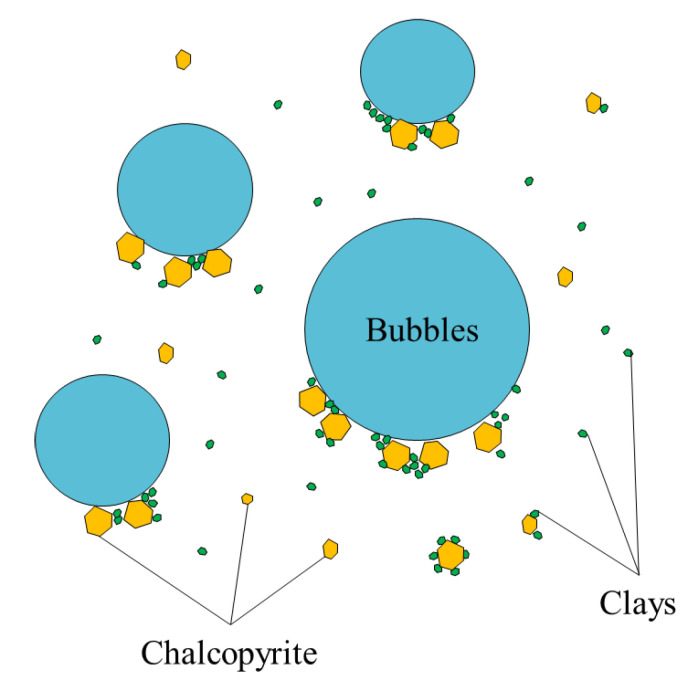
Adhesion of hydrophobized mineral and clays to a bubble.

Commonly, the collectors are not selective enough to the valuable mineral, and a significant portion adheres to gangue minerals, especially sulfur species such as pyrite, which usually have a significant presence in copper deposits [14,21,22] or phyllosilicates like clays [23]. This reduces the efficiency of the reagent and contaminates the concentrate, since all the particles covered by collectors (valuable and non-valuable) increase their hydrophobicity, favoring their adsorption to the bubbles (Figure 6).

**Figure 7:**
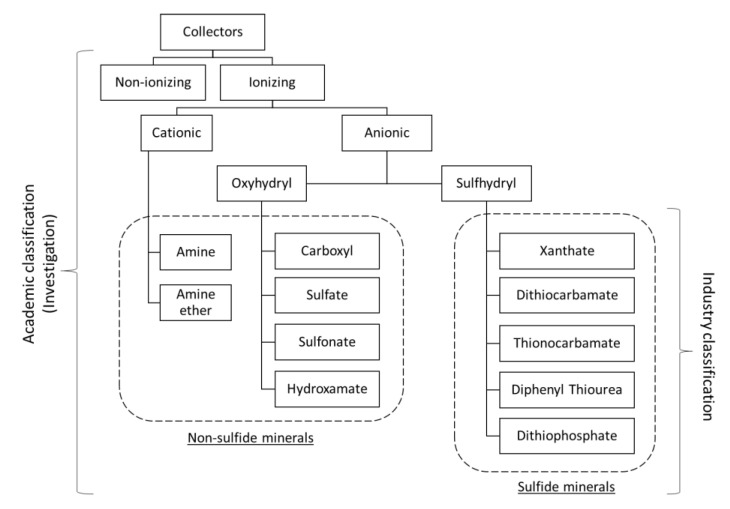
Classification of collectors according to their academic and industrial use.

For the flotation of oxides, it is typical for the hydrocarbon chain to contain between 10 to 18 CH, CH_2_, and CH_3_ groups, while in most sulfide minerals, a shorter hydrocarbon chain is contemplated. In the latter case, collector adsorption occurs predominantly by chemisorption and surface precipitation, while electrostatic mechanisms are less significant. The range of collectors used in the industry is diverse. It is based on chemical differences in the molecule, especially its functionality, where families with hydroxyl and sulfhydryl groups stand out. The latter are mainly used for sulfide minerals (see Figure 7).

Nonionic collectors are nonpolar structures, where the molecules are single chain hydrocarbons, insoluble in water. Its use is recurrent in the flotation of coal, graphite, sulfur, molybdenite, silicates, and quartz [24,25,26,27,28]. For their part, ionic collectors are the largest group of reagents. They are divided into anionic and cationic, being the charged functional group that adheres to the surfaces.

**Figure 8:**
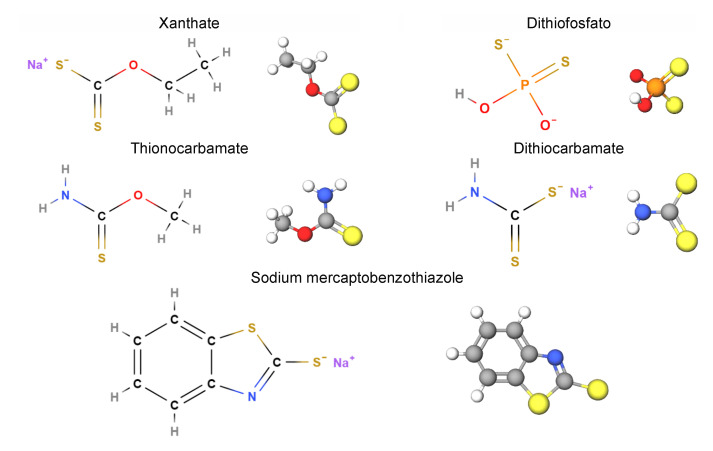
Examples of typical thiol collectors.

The most common copper sulfide flotation collectors are the hydrogen sulfide (-SH) class, also known as thiols, sulfhydryl group, or mercapto group (C-S). They can be grouped into the families of xanthate (X), dithiophosphate (DTP), dithiocarbamates (DTC), thionocarbamate, thiocarbamate, and mercapto benzothiazole (MBT) (Figure 8).

Thiol collectors have a sulfur atom in the polar group that is not bound to oxygen. Its general chemical formula is ROCS_2_M, where R is an alkyl group and M is a metal. In solution, this reagent dissociates, leaving the ROCS^2−^ xanthate ion as the active ion, and when reacting and adsorbed on the surface, it changes the affinity of the mineral with water by exposing the hydrocarbon chain. This class of collectors is characterized by its high chemical activity towards metal ions, little surface activity at the liquid–air interface, and the absence of aggregation in solution (there is no formation of micelles) [29]. However, they have the limitation that their carbon chains are short, and the adsorption process in the mineral can become inefficient. 

### 3.2. Effect of pH on Collector Behavior

**Figure 9:**
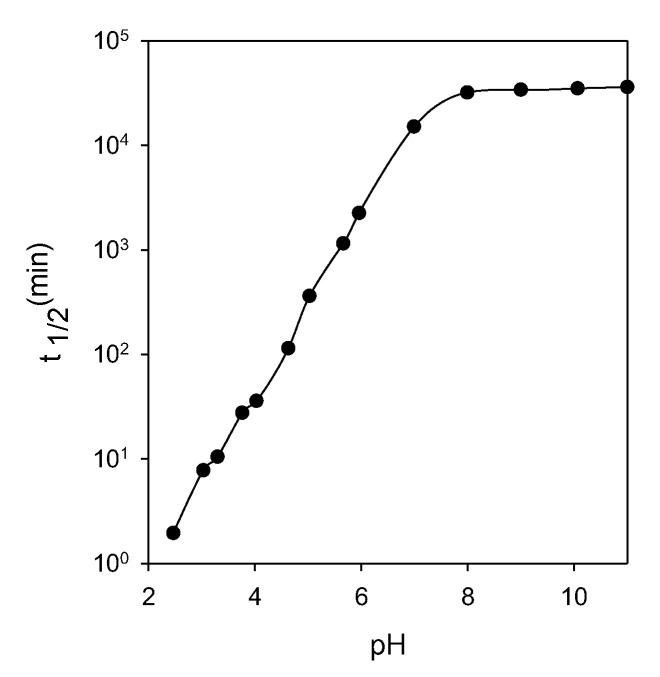
The half-life time of ethyl xanthate as a function of pH (adapted from Allison [32]).

**Figure 10:**
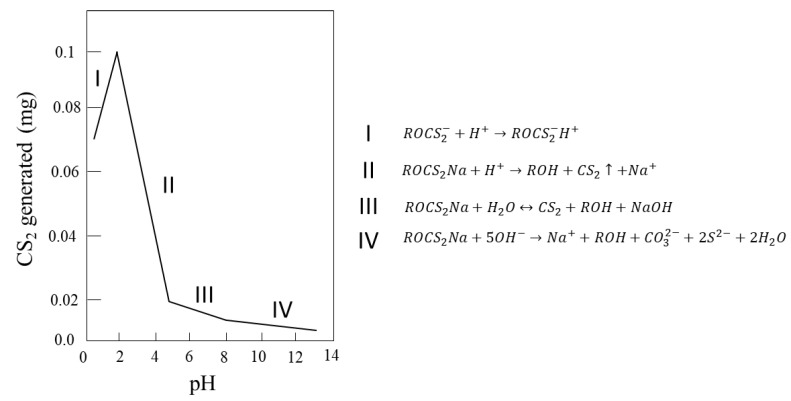
Formation of carbon sulfide at different pH values from 0.05 wt% SIBX solution (adapted from Shen et al. [33]).

Another peculiarity is that they can only be used at alkaline pH, so their implementation in leachate residues is complicated [30]. Xanthates are unstable at low pH values (Figure 9), so they are not used in acidic environments as they would produce carbon sulfide plus alcohol. Xanthates also degrade in an alkaline medium, where the dixanthogenic compound reacts with the OH^−^ anions, forming xanthate and carbon sulfide (Figure 10). These reagents are available with alkyl chains ranging from C_2_ to C_6_ (or higher) of any isomeric type [31]. Typical alkyl chains include ethyl, isopropyl, isobutyl, and amyl.

**Figure 11:**
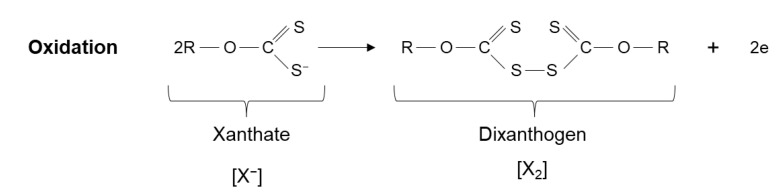
Formation of dixanthogen.

Xanthate collector forms an insoluble and hydrophobic compound with polyvalent metals (metallic xanthate compounds), and its oxidation can form dixanthogen (see Figure 11). Dixanthogen is an insoluble and highly hydrophobic oil that adsorbs on sulfurous surfaces, improving their floatability.

According to the literature, the most common mechanism of interaction between xanthate and sulfides is electrochemical [34]. Xanthate ions are oxidized to dixanthogen or metal xanthate. Dioxanthogen is not a selective reagent, unlike sulfur, which has a high affinity for copper.

**Figure 12:**
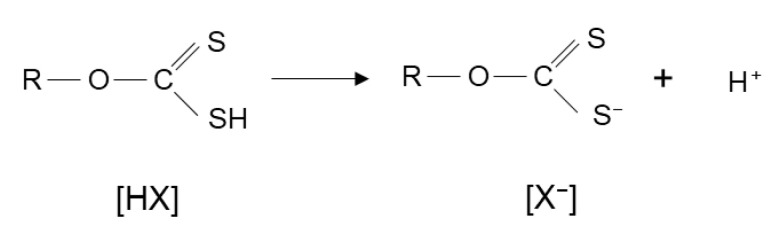
Chemical structures of xanthates. [HX]: xanthic acid, [X^−^]: xanthate anion. R: hydrocarbon group (adapted from Somasundaran et al. [35]).

Xanthates have a heteropolar molecular structure, with a non-polar carbon chain and a polar hydrogen sulfide group (-SH) that dissociates in water to give the xanthate ion and H^+^ as a product (Figure 12) [35]. The H^+^ can be substituted for sodium or potassium.

Different routes may result in the decomposition of xanthate in water, thus generating the various species mentioned previously, with six reactions having been identified [36]:

Hydrolysis of the xanthate ion and formation of xanthic acid

(3) $$K^{+}+ROCS_{2}^{-}+H_{2}O\to K^{+}+OH^{-}+ROCS_{2}H\;$$

Decomposition of xanthic acid into alcohol and carbon disulfide

(4) $$ROCS_{2}H\to ROH+CS_{2}\;$$

Hydrolytic decomposition of the xanthate ion

(5) $$6ROCS_{2}^{-}+3H_{2}O\to 6ROH+CO_{3}^{2-}+3CS_{2}+2CS_{3}^{2-}\;$$

Dixanthogen oxidation

(6) $$ROCS_{2}^{-}+\frac{1}{2}O_{2}+H_{2}O\to {\left(ROCS_{2}\right)}_{2}+2OH^{-}\;$$

Monothiocarbonate oxidation

(7) $$ROCS_{2}^{-}+\frac{1}{2}O_{2}\to ROCOS^{-}+S{}^{\circ}\;$$

Oxidation to perxanthate

(8) $$ROCS_{2}^{-}+H_{2}O_{2}\to ROCS_{2}O^{-}+H_{2}O\;$$

Equations (3) and (4) occur in acidic environments, where the xanthate is hydrolyzed at pH < 7. If it drops to pH = 2, the xanthic acid decomposes irreversibly, resulting in carbon disulfide and alcohol. In alkaline environments, Equation (5) appears, giving rise to stable products such as carbonate, hydrosulfide, trithiocarbonate, and alcohol. These reactions indicate possible dissociation products of the chemical reactions that xanthate undergoes in freshwater solutions. However, an interesting issue is to analyze the consequences in saline environments, such as seawater, due to the continuous development of mining projects that consider low-quality waters. Cruz et al.’s recently published critical review [37] describes the chemical phenomena that dominate chalcopyrite’s flotation process in seawater.

Xanthate collectors and their derivatives are the main reagents used in copper mineral flotation, especially in Cu and disseminated Cu–Mo plants. Their solubility in water tends to decrease with the increase of the hydrocarbon chain, and they usually dissociate, depending on the pulp conditions, in many xanthate species such as xanthate ions (ROCS_2_), monothiocarbonate (ROCOS^−^), xanthic acid (ROCS_2_H), carbon disulfide (CS_2_) and dixanthogen (ROCS_2_S_2_COR). Different studies have shown that the stability of xanthates in solution is affected by the alkyl group of the molecule [38,39]. Mixtures of these reagents are commonly used to favor the potential advantages of each one, according to its specification and chemical structure. Some benefits of combined use may include improvements in: (i) floatability rate [31,40], (ii) coarse particle recovery [41], (iii) dosing requirements [40,42], and (iv) grade of concentrate and recovery [43].

**Figure 13:**
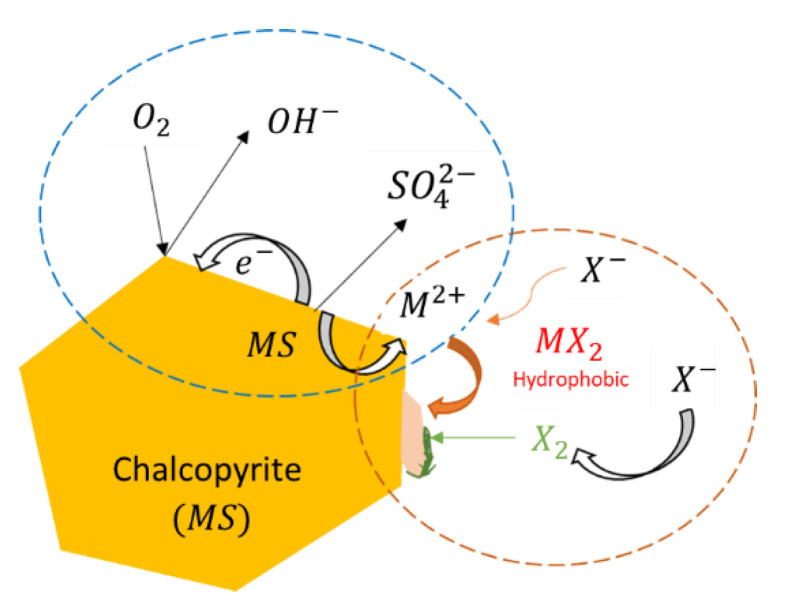
Schematization of xanthate chemical adsorption and hydrophobic coating of a chalcopyrite mineral particle (adapted from Yepsen [44]).

The adsorption of xanthates on the mineral surfaces can occur by three mechanisms: physical adsorption, chemical adsorption, and oxidation of xanthate to dixanthogen. Physical adsorption occurs at low redox potential and in the absence of oxygen, where xanthate anions [X^−^] are attracted to available sites on the mineral surface, with a positive charge density. The chemical adsorption occurs when the functional groups of the mineral surfaces react with species dissolved in the liquid phase. With slightly oxidizing conditions and oxygen, the metal sulfide surfaces oxidize to produce species such as cations, metal oxides, sulfates, thiosulfate, elemental sulfur, etc. These can generate a series of redox reactions due to the mobility of electrons at the surface level, favoring the interaction between xanthates with the surfaces’ metal cations (Figure 13).

The last mechanism occurs at specific pulp’s redox and minerals’ surface potentials, leading to oxidation of the xanthate anion [X^−^], producing dixanthogen. This organic and water-insoluble compound has been widely discussed, concluding that it is adsorbed on areas covered by MX_2_. This makes it possible to increase the hydrophobic properties of the particles significantly. In this case, the adsorption is produced by hydrophobic forces. Xanthate also dissociates to form xanthonic acid ions, thus involving the interaction of copper ions, xanthonic acid, and the surface of chalcopyrite [45]. This phenomenon occurs in two stages: (i) the chemical reaction in solution, where the copper ions react to form the Cu-xanthate complex, and (ii) the adsorption or chemical reaction on the mineral surface. The copper ions react with the chalcopyrite surface to form CuS, and the xanthonic acid ions are adsorbed onto the Cu.

**Figure 14:**
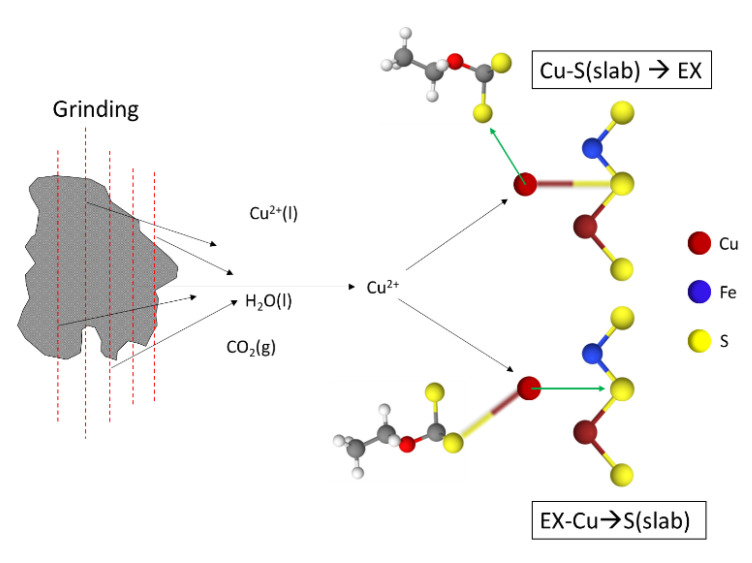
Induced activation of the flotation pathways of the copper ions released by the inclusion of fluids. EX = Ethyl xanthate (adapted from Wen et al. [45]).

The particle size reduction during the milling process gives rise to many types of surfaces with high activity, which, together with the release or inclusions of active components such as Cu, can strongly interact. Figure 14 shows two stages of the induced activation of copper ions released by fluid inclusions that, during mineral formation, were captured and lodged in microcracks during mineral growth. Conversely, copper ions react with the collector ions to form copper xanthate, adsorbed on the S surface to form a hydrophobic surface. On the other hand, copper ions also interact with the S surface to form Cu_x_S_y_, and the collector ions interact with these Cu-based sulfides [45].

Rao (2003) described the adsorption of xanthate on sulfide minerals through an electrochemical oxidation–reduction reaction occurring on the mineral surface and through a mixed potential mechanism that represents the following reactions: (i) chemisorption oxidative (Equation (9)); (ii) oxidation between the thiolate ion (xanthate) and a metal-rich surface forming a metal thiolate (metal xanthate) (Equation (10)); and (iii) formation of a dithiolate by physical adsorption on the mineral surface (Equation (11)). Equation (12) counteracts the cathodic reduction of oxygen.

(9) $$X_{ads}+e^{-}\to X^{-}\;$$

(10) $$M^{2+}+2X^{-}\to MX_{2}\;$$

(11) $$X_{2\;ads}+2e^{-}\to 2X^{-}\;$$

(12) $$O_{2}+2H_{2}O+4e^{-}\to 4OH^{-}\;$$

**Figure 15:**
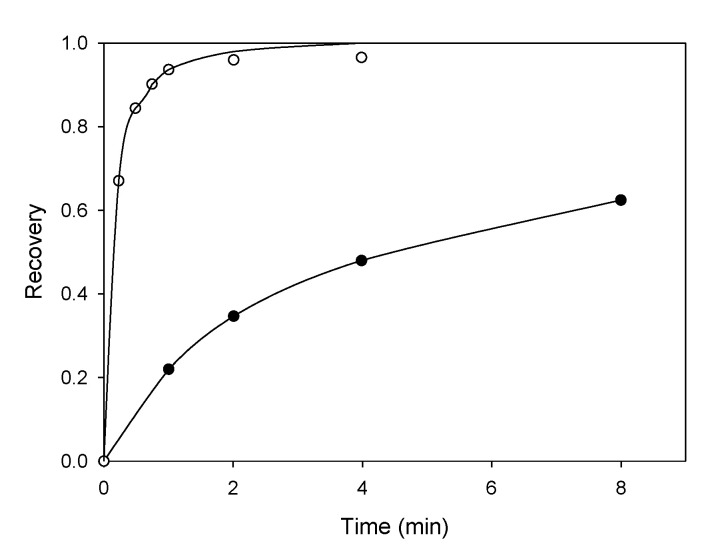
Recovery of chalcopyrite vs. flotation time without collector (black points) and 100 g/t PAX (white points). The pulp was kept at pH 9 with KOH and through an airflow of 10 mL min^−1^. The particles were conditioned for 5 min before introducing the air into the column (adapted from Vizcarra et al. [46]).

Figure 15 shows the flotation of chalcopyrite without a collector, which is kinetically slow, but the recovery significantly improved with potassium amyl xanthate (PAX) [46]. This flotation occurs from the oxidation of surface S^2−^ to S^0^, where elemental sulfur is naturally hydrophobic. The surface layers can induce the adhesion of chalcopyrite to bubbles in the absence of a collector [47].

**Figure 16:**
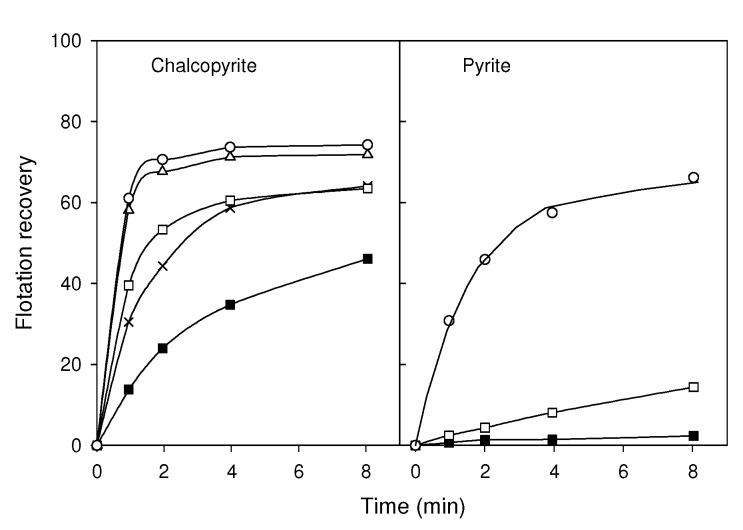
Experimental (symbols) and calculated (lines) flotation of chalcopyrite and pyrite as a function of flotation time and IBECTC (*O*-isobutyl-Nethoxycarbonyl thionocarbamate) concentration at (■) 0, (+) 5 × 10^−6^, (□) 10^−5^, (∆) 5 × 10^−5^ and (○) 10^−4^ mol dm^−3^ (2 g dm^−3^ of mineral). Water pretreated at pH 7 with 0.01 mol dm^−3^ of KCl as electrolyte and floated with nitrogen (adapted from Fairthorne et al. [14]).

The study of Fairthorne et al. [14] indicated that the flotation of chalcopyrite and pyrite at pH 7 increased with the concentration of *O*-isobutyl-Nethoxycarbonyl thionocarbamate (IBECTC) and that the flotation rate was faster for chalcopyrite. Additionally, the flotation increased with the concentration of the collector (Figure 16).

**Figure 17:**
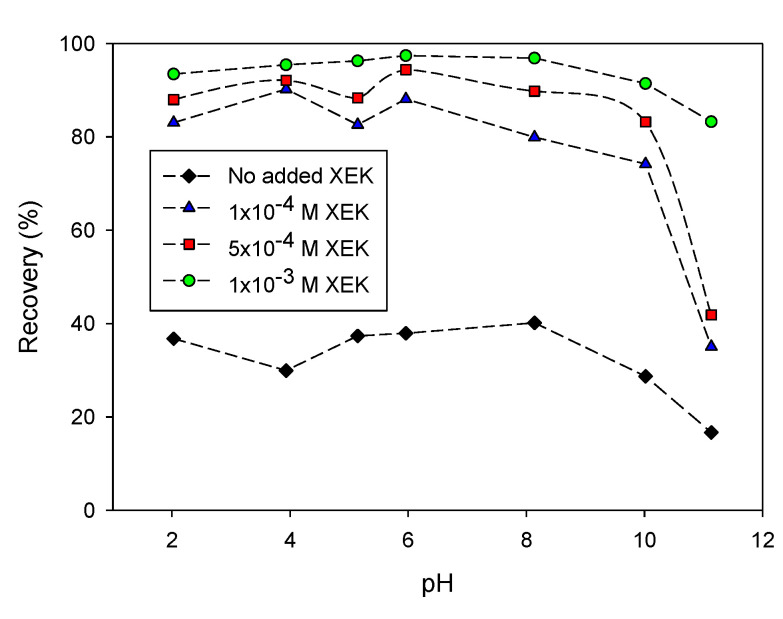
Flotation recovery of pyrite as a function of pH in the presence of various additions of potassium ethyl xanthate (adapted from Cabrera Tejeda [48]).

Cabrera Tejeda [48] analyzed the floatability of the chalcopyrite mineral as a function of pH with various dosages of ethyl potassium xanthate (XEK) (Figure 17). A drastic decrease in recovery was observed at pH > 12. At a lower pH, copper xanthate and dixanthogen formation on the chalcopyrite surface was favored, but, above pH 12, the appearance of hydrophilic species of copper hydroxide and iron is primarily favorable [49].

**Figure 18:**
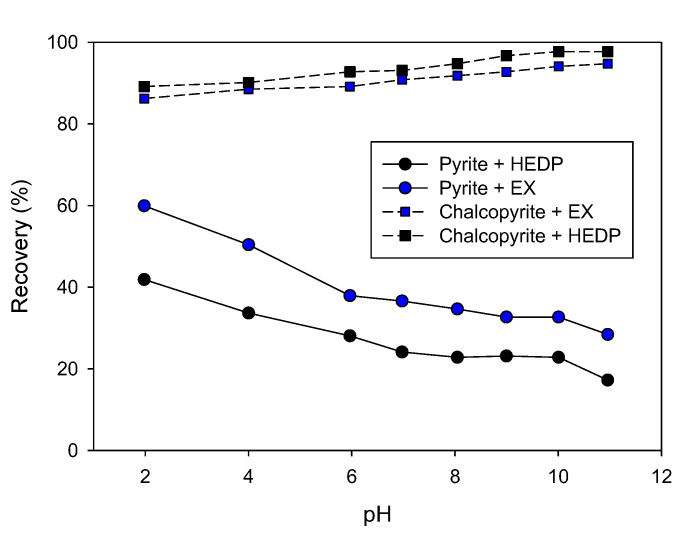
Flotation recoveries of chalcopyrite and pyrite as a function of pH using 2 × 10^−4^ mol/L of HEDP or 2 × 10^−4^ mol/L of EX as collector (adapted from Huang et al. [50]).

Ethyl xanthate (EX) is a widely used collector in the flotation of sulfide minerals. However, it also has the disadvantages of low selectivity and harm to human health. Designing more selective and green collectors is a ‘hot topic’ in mineral processing. Huang et al. [50] applied 1-Hydroxyethylidene-1, 1-diphosphonic acid (HEDP) for the first time in the flotation separation of chalcopyrite from pyrite. As shown in Figure 18, this cheap green reagent possessed a better selectivity for separating chalcopyrite from pyrite than EX.

More recent studies have included computational analyzes like density functional theory (DFT) to complement experimental tests. Mkhonto et al. [51] investigated the adsorption mechanisms of *O*-isopropyl-*N*-ethyl-thionocarbamate (IPETC), *O*-isopropyl-*N*-diethyl-thionocarbamate (IPDETC), and *S*-allyl-*N*-diethyl-dithiocarbamate (ADEDTC) on chalcopyrite reconstructed (1 1 2) surface and pyrite (1 0 0) surface. The obtained information was very interesting, since the experimental microflotation studies were linked with fundamental explanations from DFT. For example, the microflotation recoveries showed that the ADEDTC, IPETC, and IPDETC collectors gave higher chalcopyrite recoveries above 90%. The pyrite recoveries reached only 25%, with ADEDTC exhibiting the highest recoveries of the three collectors at a pH of approximately 9.0. ADEDTC was a better collector for selective flotation separation of chalcopyrite from pyrite. The authors highlighted that the ability to donate electrons decreases in the order IPDETC > IPETC > ADEDTC, and the ability to accept electrons decreases in the order ADEDTC > IPETC > IPDETC. This implied that ADEDTC, IPETC, and IPDETC could react with the surface of copper sulfide minerals through regular covalent bonds and back donation covalent bonds. The adsorption of the thiocarbamate collectors was observed to occur preferentially on Cu atoms over Fe atoms. This resulted in the order of thiocarbamate collector adsorption on chalcopyrite Cu sites decreasing as ADEDTC > IPDETC > IPETC, which showed that ADEDTC exhibits the strongest exothermic adsorption. The interaction of the thiocarbamate collectors on pyrite was weaker than the adsorption on chalcopyrite, and the order of adsorption strength decreased as ADEDTC > IPETC > IPDETC.

There are a wide variety of studies that delve into the use of different types of collectors on chalcopyrite flotation. Table 1 summarizes the main published articles.

## 4. pH Modifiers

**Figure 19:**
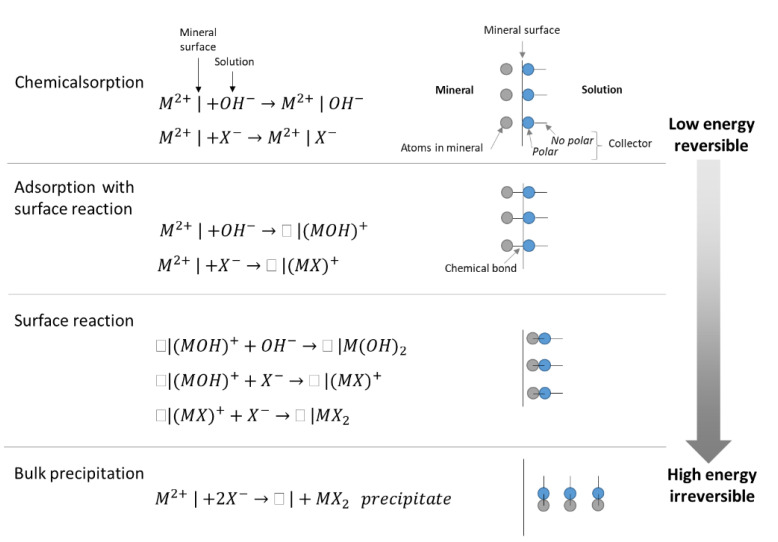
Mechanisms of interaction between the sulfide mineral surface and the collector. Both oxyhydric and sulfhydric ions interact with the mineral surface, competing for adsorption (adapted from Zanin et al. [79]).

With rare exceptions, the effectiveness of flotation reagents strongly depends on the pulp’s pH. Consequently, one of the fundamental objectives is to elucidate the optimum pH for a specific combination of reagents and minerals. The H^+^ and OH^−^ ions compete with other ions present in the solution to reach the mineral surfaces, influencing the salts’ dissociation and ionic exchange. As observed in Figure 19, if a reactant or species is attracted by chemisorption, it will only reach the coverage of the monolayer and will be able to bind with an ion on the mineral surface; but it will not be able to react. However, if adsorbed, the species will displace the lattice ion from the mineral surface. This process is called adsorption with surface reaction, and can occur with hydrolyzed species, as shown in the reactions.

**Figure 20:**
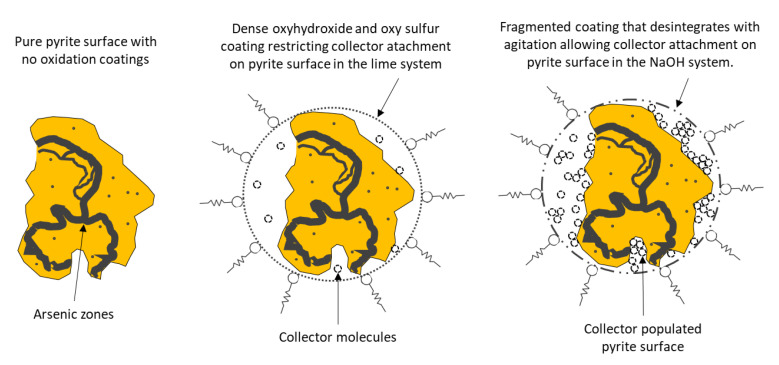
Schematic representation of the pyrite surfaces without treatment (**left**), with lime treatment (**center**), and with NaOH treatment (**right**) (adapted from John et al. [80]).

In general, sulfur mineral plants operate under alkaline conditions. Commonly used pH regulators include lime and sodium hydroxide. Due to its low cost, lime is used in almost all flotation circuits, and stabilizes goethite, sulfated oxyhydroxides, and other oxidation products on the surface of pyrite [80]. However, studies have shown that lime tends to form a dense, stable, and impermeable layer on the surface of the pyrite ore that prevents collectors from reaching and hydrophobizing it, and even increasing its size with longer conditioning time [81,82,83]. Schematized in Figure 20, this layer is the product of the strong cohesion that the Ca^2+^ ions exert, providing the solution with high ionic strength. On the other hand, the use of NaOH does not show this behavior on the pyrite surface [83].

If a chalcopyrite flotation plant works at pH close to 11, the pH being modified by lime, it is expected that the collector will selectively adsorb on the chalcopyrite surface. If the reservoir contains pyrite, it will likely not float excessively. The change in pH allows the copper and iron atoms to dissolve from the copper ore lattice, leaving a surface deficient in metals and rich in sulfur rather than elemental sulfur (Equation (13)).

(13) $$CuFeS_{2}\;\overset{\begin{array}{c} H^{+}\\ H_{2}O \end{array}}{\to }\;{\left(CuS\right)}_{1-y}{\left(FeS\right)}_{1-z}{\left(S^{2-}\right)}_{y+z}+yCu^{+}+zFe^{3+}\;$$

Chalcopyrite floats properly with a moderate concentration of xanthate over a wide pH range; therefore, it is common for the main proposed mechanism to consider the formation of dixanthogen (Figure 11) [84,85] and, in some cases, the formation of cuprous xanthate [86,87]. While both species can form simultaneously, the proportions vary depending on the carbon chain length. It has been concluded that the optimal chain length is between the C_5_–C_8_ range [88].

Metal xanthate (MX) or dixanthogen (X_2_) acts as hydrophobic species [89]. However, the selectivity of dixanthogen is better than that of xanthate. The main reason for this lies in its steric effect (size), considering that the molecule’s structure is larger than that of xanthate [90]. For example, in the flotation of chalcopyrite and galena minerals, the xanthate-K_2_Cr_2_O_7_ depressant pair showed low recovery instead of using the dixanthogen-K_2_Cr_2_O_7_ depressant, where the separation results are much better [90].

The chalcopyrite flotation mechanism also takes into consideration the chemisorption of xanthate on the mineral surface. This is verified by considering that the dixanthogen is not stable at pH values above 11; however, Fuerstenau et al. [91] showed that in the presence of ethyl xanthate, the floatability of chalcopyrite was between pH 3–12. If the only mechanism responsible for flotation is xanthate oxidation, a depression at pH 11 would be expected.

**Figure 21:**
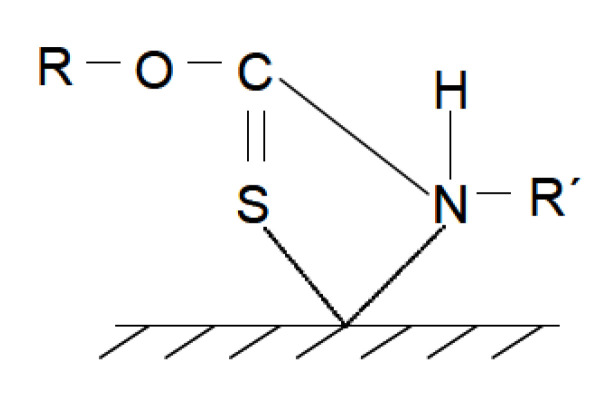
Schematic representation of the interaction of the thionocarbamate collector with copper atoms on the chalcopyrite surface.

Other collectors used in chalcopyrite flotation include dithiocarbamates and thionocarbamates, which operate over a wide pH range (pH 5–9.5). These collectors can have a greater recovery when decreasing the pH, and have high specificity for copper ions [14]. Due to the introduction of groups that accept electrons, the interaction of S atoms and Fe ions decreases, but the interaction of Cu continues to be maintained, and the selectivity of the collector is reasonable [90]. The adsorption of thionocarbamates on the surface of chalcopyrite is illustrated in Figure 21, where the chelating agents N, O, and S interact more with cuprous ions than with ferric ions [92,93,94]. The thiocarbamate selectivity decreases with increasing R and R′ [95].

Dithiophosphates are useful for copper sulfide flotation at pH > 9 [96,97,98] and are commonly used in supplemental cleaning circuits, as they are more selective to copper sulfides (from the iron) than xanthates. The interaction mechanism is believed to be through chemical adsorption, through the formation of cuprous dithiophosphate under neutral and slightly alkaline conditions [99]. Dithiophosphate, which is also known as Aeroflot, has a collection performance that is somewhat weaker than xanthate. However, its selectivity and stability may be more attractive, and it has also been found to favor the froth quality [100]. It is the most widely used reagent among the various Aeroflot-type collectors, and has an excellent capacity for collecting chalcopyrite, galena, and sphalerite. Some disadvantages are its corrosiveness and toxicity, and it can cause certain types of contamination; therefore, its use should be moderate. In addition, the use of small amounts of a dithiophosphate-type collector (secondary collector) and xanthate (primary collector) offer more significant metal recovery in bulk recoveries.

Based on their chemical nature, collectors have a pH range in which they can operate efficiently, where their molecular structure remains stable (Table 2). The critical pH also depends on the surface characteristics of the mineral, as reported in Table 3.

**Figure 22:**
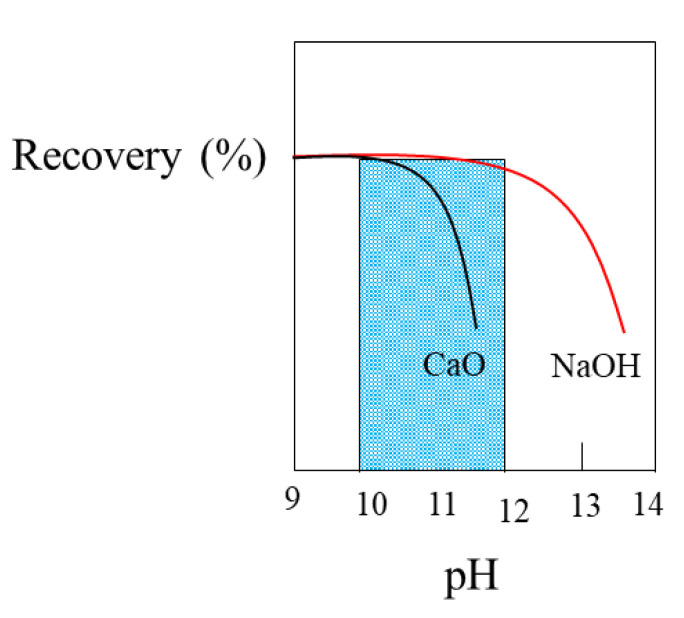
Recovery of pyrite with lime and caustic soda (adapted from Coloma Álvarez [102]).

Pyrite’s flotation range is between pH 3 and 10.5. Interestingly, this limit is related to the oxidation of xanthate, above which there is no formation of dixanthogen. This result is consistent with the study by Richardson et al. [101], who showed that dixanthogen is the main promoter species of pyrite flotation. It should be noted that pyrite depression is favored by Ca^2+^ ions since hydrophilic complexes are formed at alkaline pHs, which adhere to the mineral surface, reducing its affinity with bubbles. For this reason, sodium hydroxide (NaOH) does not have the same depressant effect as lime (Figure 22), as will be discussed in Section 5.4.

## 5. Pyrite Mineral in the Presence of Chalcopyrite

Pyrite, being the most abundant iron sulfide mineral, is commonly associated as a gangue mineral from base metals such as copper, lead, and zinc [103,104,105,106,107,108] and in copper minerals of hydrothermal origin. This mineral is naturally floatable, and under certain conditions, it can be recovered even without the use of collectors [109].

### 5.1. Pyrite Flotation

Pyrite has poor floatability under alkaline conditions [85]. However, it is possible to obtain high recoveries with standard collectors of the hydrogen sulfide type, such as xanthates, dithiophosphates, and carbamates [31]. When using xanthate, the flotation of pyrite is affected by electrochemical reactions that are the product of the oxidation of both the collector and the mineral itself [107,110]. The oxidation of sulfides arises from the interaction of species found on the mineral surface with water and oxygen. Under acidic conditions, metal ions migrate to the surface to dissolve in solution, leaving a surface rich in sulfur (Equation (14)).

(14) $$MS+2nH^{+}+\frac{n}{2}O_{2}\overset{\;}{↔}M_{1-n}S+nM^{2+}+nH_{2}O\;$$

Under alkaline conditions, these ions hydrolyze and form a metal hydroxide layer, a hydrophilic chemical species (Equation (15)).

(15) $$MS+\frac{1}{2}O_{2}+H_{2}O\overset{\;}{↔}M_{1-n}S+nM{\left(OH\right)}_{2}\;$$

**Figure 23:**
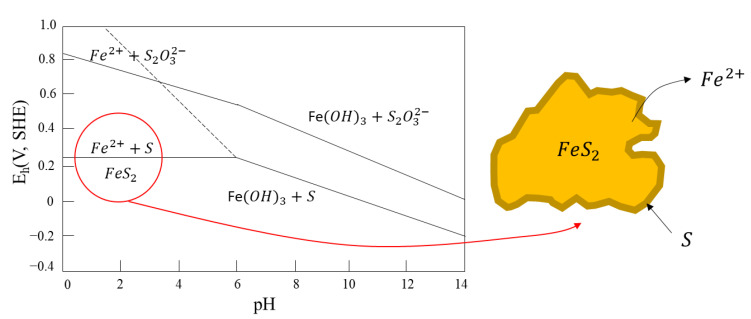
Eh-pH diagram for pyrite in aqueous solutions, with elemental sulfide as a meta-stable phase. The equilibrium lines correspond to species dissolved at 10^−4^ mol/L (adapted from Hu et al. [111]).

As shown in Figure 23, a surface rich in sulfides, depending on the degree of oxidation, may have low metal sulfide (M_1−n_S), polysulfide (S_n_^2−^), or elemental sulfide (S^0^), which will promote hydrophobicity and could eventually induce flotation in the absence of a collector (Equations (16) and (17)).

(16) $$\;FeS_{2}\to Fe^{2+}+2S+2e^{-}\;\;E_{h}=0.24\;V$$

(17) $$FeS_{2}+3H_{2}O\to Fe{\left(OH\right)}_{3}+2S+3H^{+}+3e^{-}\;\;E_{h}=(0.579–0.059\;\mathrm{pH})\;V$$

On the other hand, generation of metal hydroxides and sulfates is also likely to occur, which are hydrophilic species that favor pyrite depression, which arises in Equations (18) and (19).

(18) $$FeS_{2}+3H_{2}O\to Fe^{2+}+S_{2}O_{3}^{2-}+6H^{+}+6e^{-}\;\;E_{h}=(0.344–0.059\;\mathrm{pH})\;V$$

(19) $$FeS_{2}+6H_{2}O\to Fe{\left(OH\right)}_{3}+S_{2}O_{3}^{2-}+9H^{+}+7e^{-}\;\;E_{h}=(0.48–0.076\;\mathrm{pH})\;V$$

**Figure 24:**
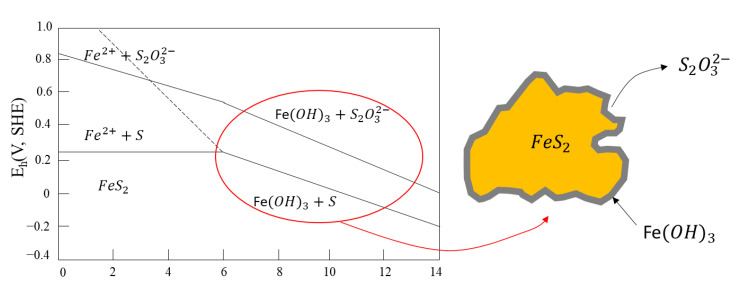
Eh-pH diagram for pyrite in aqueous solutions shows the metal hydroxide and sulfate formation zone. The equilibrium lines correspond to species dissolved at 10^−4^ mol/L (adapted from Hu et al. [111].

An Eh-pH diagram for pyrite in aqueous solutions is shown in Figure 24. The species within the red line denote the conditions in which pyrite flotation is observed without collectors. Accordingly, pyrite flotation can only be achieved at ranges of pH below 5 and potential below the continuous upper line without the formation of thiosulfate (S_2_O_3_^2−^) or iron hydroxide (Fe(OH)_3_). At higher pHs and moderate potentials (0–100 mV), the generation of metal hydroxides and sulfates (hydrophilic species) occurs, causing a sulfide depression.

However, the presence of thiosulfates is thermodynamically unstable and tends to be disproportionate to S^2−^ and SO_4_^2−^. Therefore, their stability is determined by the thermodynamic activities of the ions in the solution.

An essential parameter is the resting potential of the minerals in the solution. This is related to contact with the electrolyte (the process water), which induces a galvanic interaction [36,112]. Table 4 shows that the resting potential of pyrite (0.22 V) is greater than the reversible potential of dixanthogen (0.13 V). In the presence of xanthates, the formation of dixanthogen is favored on the surface of pyrite [18,113,114].

**Figure 25:**
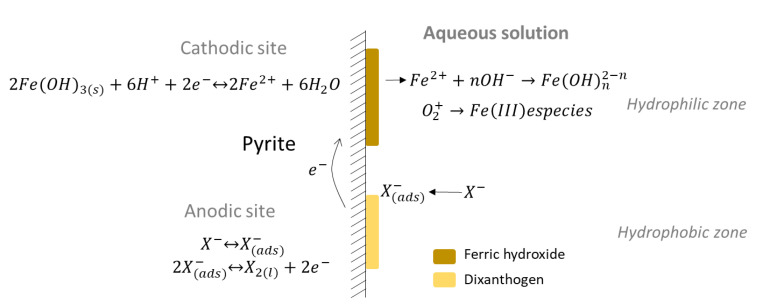
Adsorption and formation of dixanthogens on the surface of non-activated pyrite (adapted from Valdivieso et al. [115]).

**Figure 26:**
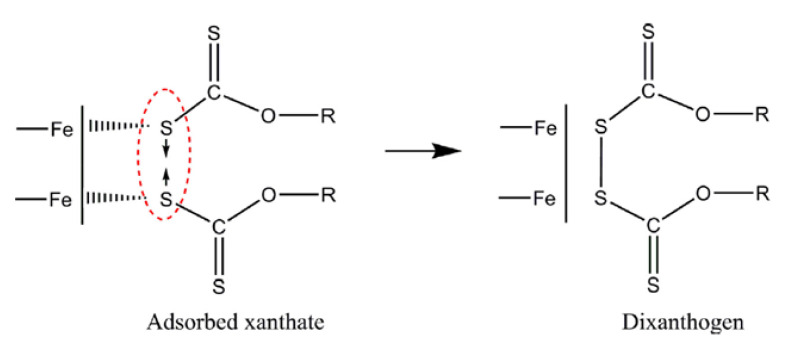
Dixanthogen formation on the surface of pyrite (adapted from Wills et al. [116]).

Pyrite, which has the highest resting potential of the common sulfides, is referred to as being the most “noble”. Xanthate adsorption can now be understood as an anodic reaction on the mineral surface resulting in the oxidation of xanthate ions to dixanthogen. Subsequently, the cathodic reaction occurs where the reduction of ferric hydroxide to Fe^2+^ ions occurs (Figure 25). The surface behaves as a catalyst for cathodic and anodic reactions in this case. According to this mechanism, as dixanthogen is formed, there is a reduction in the hydrophilic surface of pyrite (Figure 26).

### 5.2. Pyrite–Chalcopyrite Flotation

Pyrite, with the highest resting potential, acts as a cathode and is galvanically protected, while chalcopyrite, with a lower resting potential, acts as an anode [117,118,119]. This separation is difficult due to the oxidation and dissolution of chalcopyrite and subsequent activation of copper on the pyrite surface [120,121]. The main problem in separating chalcopyrite from pyrite is related to the selectivity of the collectors, a product of accidental activation caused by Cu^2+^ ions, which dissolve from the valuable mineral. This activation (detailed in Section 5.3) is also influenced by the grinding media, which modify the electrochemical conditions of the pulp [122]. The copper ions are released into the solution in a grind with chalcopyrite and pyrite minerals. They are rapidly adsorbed on the reactive sulfur sites on the surface of the pyrite, reducing the copper ions by oxidizing the sulfur.

**Figure 27:**
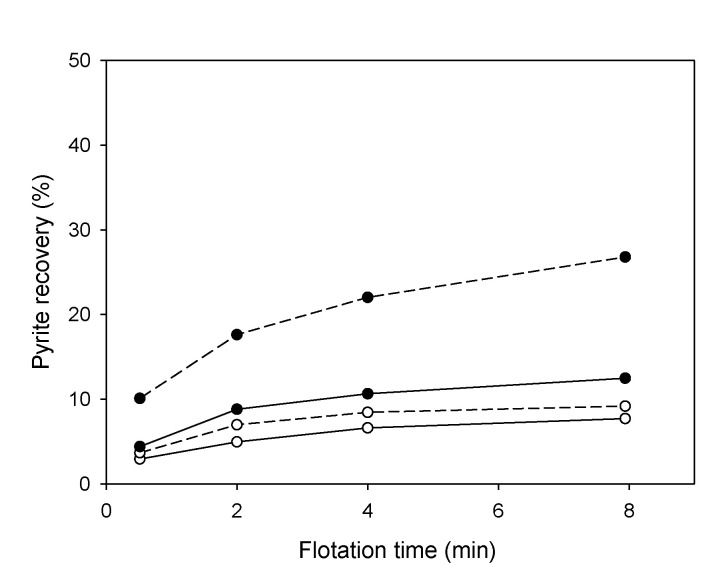
Pyrite mineral flotation as a function of time under oxidizing conditions: (o) no Cu^2+^ ions; (●) with 1.5 × 10^−3^ M Cu^2+^; (dotted lines) grinding balls in steel; (solid lines) 30 wt% of grinding balls with chromium (adapted from Peng et al. [121]).

**Figure 28:**
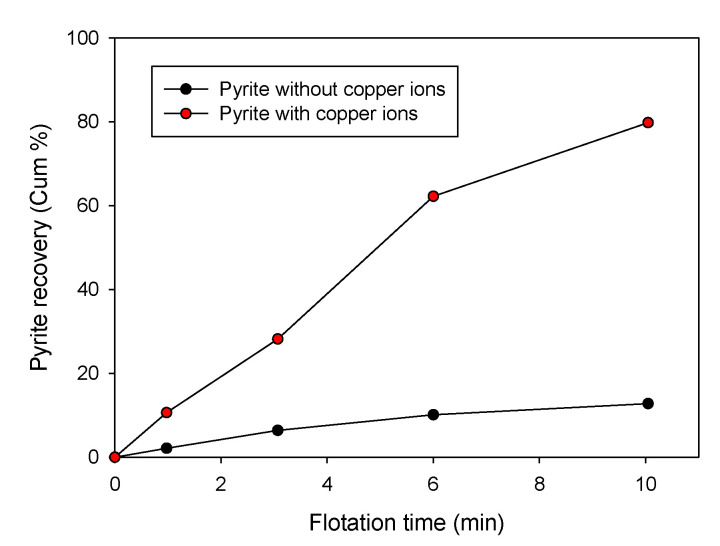
Recovery of pyrite in the absence and presence of 300 g/t CuSO_4_·5H_2_O with PAX at pH 9.0 (conditions: 1.5 L flotation cell where H_2_SO_4_/NaOH was added to adjust the pH and airflow of 6 dm^3^/min) (adapted from Mu et al. [123]).

Figure 27 presents results obtained in the study published by Peng et al. [121]. The authors showed that the flotation of pyrite is low in the absence of copper. At the same time, the increase in recovery would occur mainly due to the formation of a new phase of copper sulfide on the surface of the pyrite through the reduction of Cu (II) to Cu (I). This reduction, accompanied by oxidation of the surrounding sulfur, enhances xanthate adsorption on the pyrite surface. Similarly, Mu et al. [123] indicated that in an alkaline solution, pyrite is activated in the presence of copper ions, and its flotation increased when adding 300 g/t of CuSO_4_•5H_2_O (Figure 28).

When grinding with chromium media, higher chalcopyrite and lower pyrite recoveries are produced than with steel media. This is attributed to the fact that steel is electrochemically active and increases the formation of iron hydroxides on the surface of the copper ore. Milling under reducing conditions prevents the formation of these oxidation products and allows effective separation [124]. However, the most common is to use depressants such as polymers, hydroxides, carbonates, cyanides, or sulfites [125], as discussed in Section 5.4.

**Figure 29:**
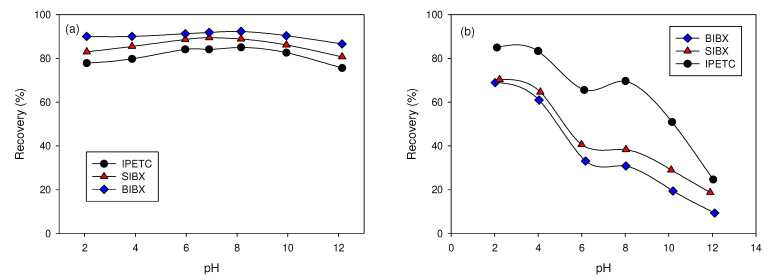
Recovery of pure chalcopyrite (**a**) and pyrite (**b**) as a function of pulp pH (collector concentration is 4 × 10^–5^ mol·L^−1^) (adapted from Ma et al. [126]).

Many studies have proposed new reagents derived from xanthate, introducing additional active sites in the molecule that allow increasing the recovery and selectivity of chalcopyrite with respect to pyrite. For example, Ma et al. [126] synthesized a new compound, *S*-benzoyl *O*-isobutyl xanthate (BIBX), from sodium isobutyl xanthate (SIBX) and benzoyl chloride. It was designed by incorporating a benzyl carbonyl group into the xanthate structure. The BIBX binds to the mineral surface through the thiol’s sulfur atoms and the carbonyl’s oxygen, exhibiting even a better collection capacity and selectivity for chalcopyrite than the SIBX, having a recovery of up to 92% of pure chalcopyrite at pH = 8 (see Figure 29a). The adsorption data indicates that BIBX exhibits a stronger affinity for chalcopyrite than pyrite under slightly acidic or alkaline conditions (see Figure 29b). Xiao et al. [127] prepared the new collector *O*-isopropyl-*S*-[2-(hydroxylamine)propyl]dithiocarbonate ester (IPXPO), which carries thion and oxime groups, and is synthesized from sodium isopropyl xanthate (SIPX). IPXPO exhibited superior flotation performance for chalcopyrite than pyrite, and was preferentially attached to chalcopyrite surfaces at pH 4–9. FTIR spectra further elucidated that the IPXPO reaction of C=S probably produced Cu surface complexes and –C=N–OH groups of IPXPO with copper atoms to form Cu–S, Cu–N, and Cu–O bonds on chalcopyrite surfaces.

### 5.3. Pyrite Activation

**Figure 30:**
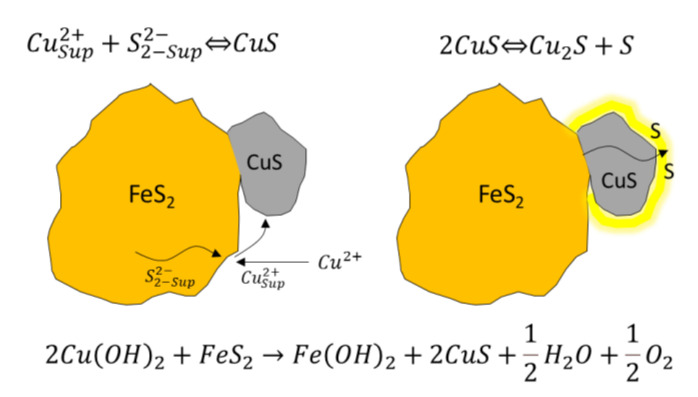
Schematization of pyrite activation by Cu^2+^ ions and subsequent sulfur formation.

The presence of copper (Cu^2+^) and lead (Pb^2+^) ions in the solution is due to the partial dissolution of minerals such as chalcopyrite and galena [121,128]. The presence of iron ions is also related to the wear suffered by the grinding media used in the previous size reduction processes [129]. The activation of pyrite by Cu^2+^ has been studied extensively, accepting that it is produced mainly by reducing copper (II) to copper (I). This is accompanied by the oxidation of sulfide ions on the pyrite surface [130,131] (Figure 30).

The following pathways are accepted to induce pyrite activation by Cu (II) ions:

1. When the mineral contains soluble copper oxides (such as chalcanthite, atacamite, etc.) dissolved in solution, they can react with other minerals or reagents.
2. Due to galvanic (electrochemical) effects during grinding and regrinding, the interaction with the steel balls or even the mill material allows the release of ions.
3. When cupric ions activate the pyrite surface, the adsorption of the collector on its surface increases, because xanthate and derivatives react strongly with copper species, forming CuX and X_2_ (dixanthogen) [132,133,134]. As a result, collector-activated pyrite floats in a moderately alkaline pH condition.

### 5.4. Pyrite Depression

The appearance of pyrite in the concentrate is problematic, since the oxidation reactions of smelting stages (after froth flotation) generate sulfur dioxide (SO_2_), which could be released into the environment. If the sulfuric acid plants cannot process all the SO_2_ generated during smelting [107]. Additionally, higher iron content in the concentrate implies a higher requirement for quartz flux to achieve good separation of the mate from the slags. This acidifies the slag, increases its viscosity, and increases copper losses due to smelting entrainment. These effects can be minimized if flotation operations generate high-quality concentrates with low pyrite content. Among the main alternatives, the following can be considered:

(i) Use of more selective collectors than xanthates such as thionocarbamates, dithiophosphates, dithiophosphinate, dithiocarbamates, xanthoformates;
(ii) Use of pyrite depressants, including inorganic reagents such as pH modifiers (OH^−^ ions), oxygen conditioning, sodium cyanide, sulfoxide depressants (such as bisulfite, sulfite, sodium metabisulfite). Extensive studies have also been reported highlighting natural polymers such as starch, dextrin, guar gum, carboxymethyl cellulose, and diethylenetriamine [107,108,135,136].

The most common method of depressing pyrite is raising the pH of the pulp (to above pH 10.5). This generally causes a notable decrease in the floatability of iron sulfide (see Figure 22), which has been established as being for the following reasons:

(i) The formation of a layer of ferric hydroxide (Fe(OH)_3_) is promoted on the surface of the pyrite, which is hydrophilic and prevents the adsorption of the collector;
(ii) The oxidation of pyrite occurs at lower potentials than the oxidation of xanthate to dixanthogen; therefore, the formation of dixanthogen is inhibited.

**Figure 31:**
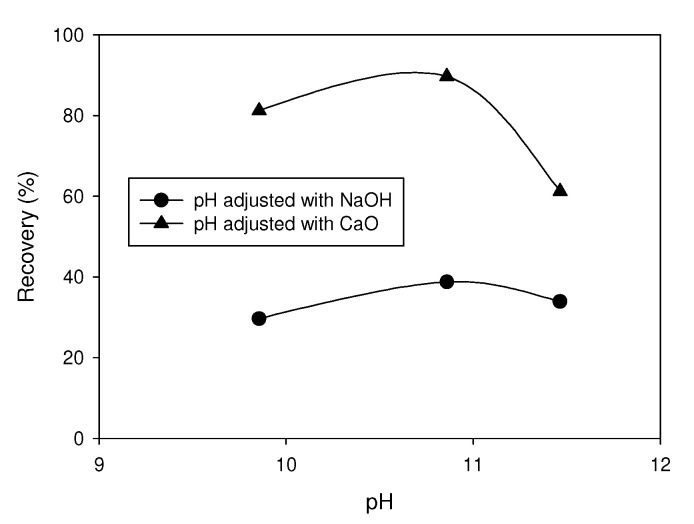
Pyrite recovery as a function of a pH adjusted with NaOH and CaO. Conditions: 1 × 10^−4^ mol/L of CuSO_4_ and 5 × 10^−5^ mol/L of butyl xanthate (adapted from Li et al. [138]).

The depression of pyrite depends on the type of pH modifier. For example, Yuqiong et al. [137] analyzed the differences generated by sodium hydroxide and lime in a system in which pyrite was activated with copper sulfate. Then, 2 g of the mineral were added to an XFGC-80 flotation cell. The reagents were added in the following order: (i) NaOH or CaO with 3 min conditioning, (ii) 1 × 10^−4^ mol/L of CuSO_4_ with 5 min conditioning, and (iii) 5 × 10^−5^ mol/L of butyl xanthate with 5 min conditioning. The flotation time was set at 3 min. Figure 31 shows that throughout the pH range, less recovery is achieved when lime is used. At a pH < 11 using NaOH, the pyrite recovery was greater than 80%, a value that dropped to 60% when the pH increased to 11.5. While using lime, the recovery was less than 40%, presenting slight variation with respect to pH.

The addition of lime increases the pH and release of calcium ions (Ca^2+^) to the solution, influencing pyrite depression by two factors [138,139,140]:

(i) Generation of hydroxide-type compounds on the surface of pyrite, which shows high hydrophilicity;
(ii) Calcium ions that are adsorbed on the pyrite surface, reducing the adsorption of dixanthogen.

### 5.5. Zinc Sulfate

**Figure 32:**
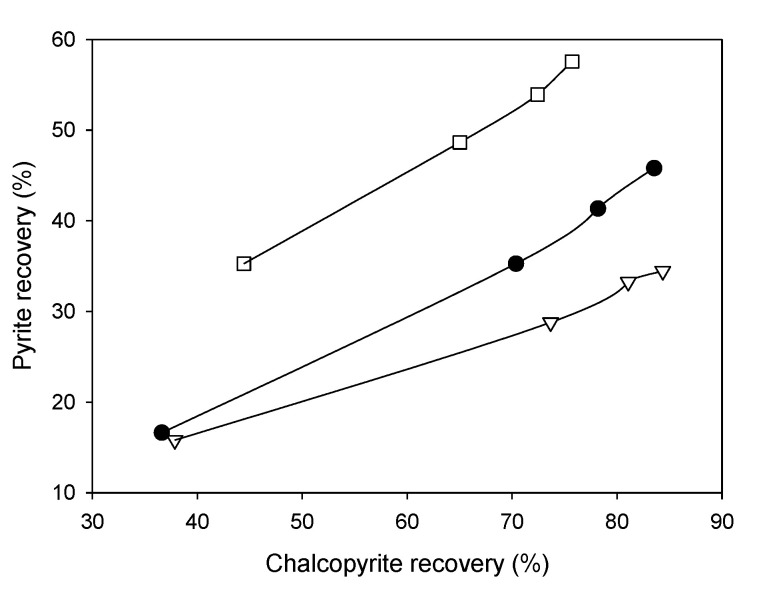
Pyrite and chalcopyrite flotation as a function of flotation time and ZnSO_4_ concentration of (□) 0 g/t, (●) 500 g/t, and (∆) 2000 g/t (pH = 9.0; [SIPX] = 200 g/t; conditioning Eh = 275 mV, SHE) (adapted from He et al. [125]).

Zinc sulfate (ZnSO_4_) has been used to separate the chalcopyrite/pyrite system, and only acts as a depressant when it is in hydroxyl ions [23]. He et al. [125] showed the recovery of pyrite and chalcopyrite as a function of flotation time and ZnSO_4_ concentration with pulp at 275 mV (SHE) (Figure 32). The addition of zinc sulfate (2000 g/t) caused, after 8 min of flotation, a decrease in the pyrite recovery from 58% to 34%. The authors suggested selective adsorption through electrostatic interactions between zinc hydroxide and ferric hydroxide under slightly alkaline conditions. Meanwhile, the recovery of chalcopyrite increased from 76% to 81%.

**Figure 33:**
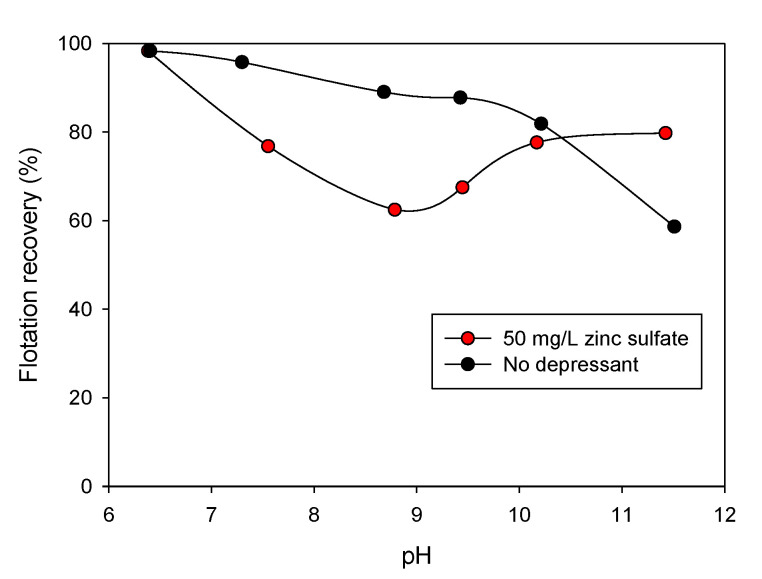
Chalcopyrite recovery vs. pH (2g with a size of −38 µm in 5 × 10^−4^ mol/L of isopropyl sodium xanthate as a collector; 200 mL of distilled water, pH fixed with NaOH or HCl and 20 mg/L DF250) (adapted from Cao et al. [142]).

Olsen et al. [141] indicated that zinc sulfate could induce the formation of sulfoxide and hydroxide species on mineral surfaces, inhibiting the collector adsorption. However, the depression of pyrite by zinc sulfate is potential dependent (Eh). The reagent does not work correctly at low Eh values, because fewer hydroxide groups form on the mineral surface that allow for the fixation of zinc hydroxide. Cao et al. [142] analyzed the recovery of chalcopyrite in 50 mg/L of zinc sulfate in columnar flotation tests, which reduced its floatability, reaching its optimum at values of up to pH 9. This reduction corresponds to 40%, where after pH 10, chalcopyrite can coagulate (Figure 33). This coagulation, caused by the added zinc sulfate, is due to the added zinc cations and the pH region. These cations go through hydrolysis reactions forming hydroxyl and zinc hydroxide species. Zinc hydroxyl and hydroxide species are known to be highly surfactant and can adsorb (zinc hydroxyl) or precipitate (zinc hydroxide) on mineral surfaces [142,143].

### 5.6. Cyanide

Cyanide is used in many mining plants, but its use has been questioned due to safety and environmental issues. This is because the xanthate and cyanide ions compete for the surface of pyrite, as occurs with the hydroxyl ion. The pyrite surface is covered by the Fe(CN)_6_^4−^ ion according to the following reaction (Equation (20)) [125]. In addition to pyrite, xanthate competes with cyanide for adsorption on the surface of chalcopyrite ore, so cyanide application is expected to reduce the buoyancy of copper ores.

(20) $$7Fe^{2+}+18HCN\to Fe_{4}{\left[Fe{\left(CN\right)}_{6}\right]}_{3}+18H^{+}+4e^{-}\;$$

The depressing effect of cyanide results from electrochemical adsorption on the pyrite, thus preventing the adsorption of the collector on its surface. Additionally, other phenomena may occur, such as dissolution of the metal xanthate, oxygen consumption, and reduction of the pulp that inhibits the adsorption of xanthate on the surface [18,116,144].

**Figure 34:**
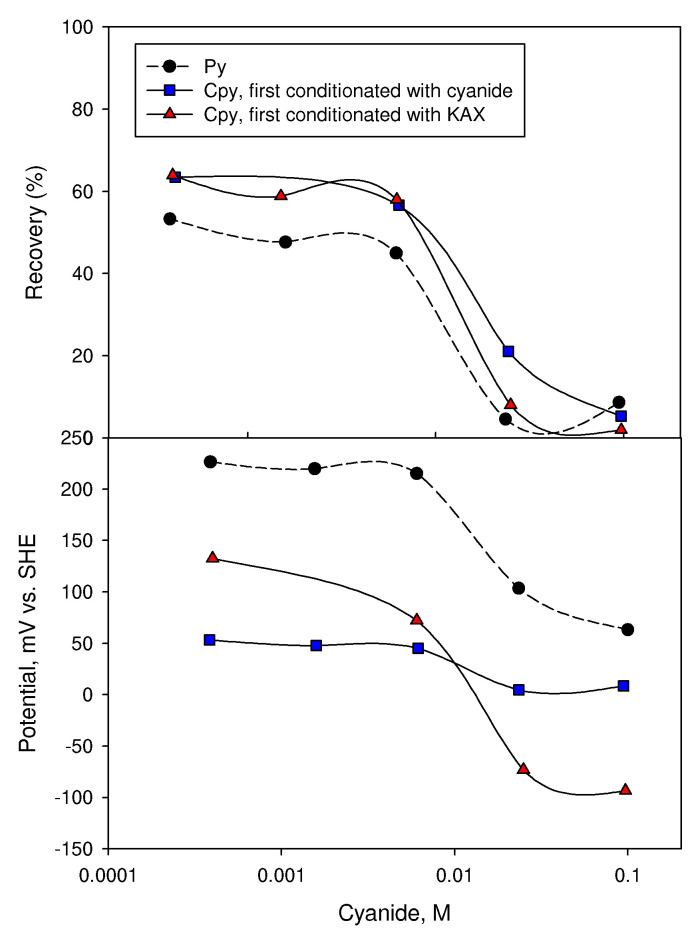
Recovery and flotation potential of pyrite and chalcopyrite through cyanide as a depressant. Conditions: potassium amyl xanthate (KAX), nitrogen use for airflow (adapted from Kocabağ et al. [145]).

The results shown in Figure 34 [145] show a critical concentration of cyanide, corresponding to a critical pulp potential above which the floatability of chalcopyrite and pyrite drops sharply.

The species sulfide and sulfites (SO_3_^2−^), bisulfite (HSO_3_^1−^), metabisulfite (S_2_O_5_^2−^), and sulfur dioxide (SO_2_) have been widely used to control the flotation of sulfide minerals at moderately alkaline pH [133,146,147,148,149,150,151,152,153]. The reducing characteristics of this type of inorganic compound inhibit the formation of the dixanthogen and its subsequent adsorption on the copper sulfide particles. Equation (21) indicates the oxidation of sulfite ions to sulfate.

(21) $$SO_{3}^{2-}+2OH^{-}\to SO_{4}^{2-}+H_{2}O+2e^{-}\;E_{0}=-0.93\;V$$

**Figure 35:**
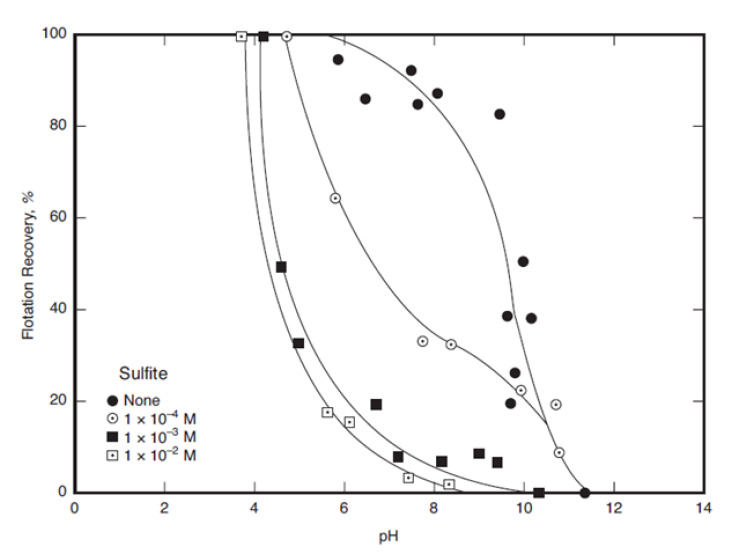
Pyrite recovery as a function of pH with 2 × 10^−4^ M ethyl xanthate in the absence and presence of sodium sulfite as a depressant (adapted from Miller [154]).

Figure 35 shows the recovery of pyrite with ethyl xanthate in the absence and presence of sodium sulfite [154]. When sulfite is not present, the pyrite depression occurs above pH 10, while with sulfite additions, the pH of the pyrite depression drops to a minimum of pH 5 (10^−3^ M).

### 5.7. Sodium Metabisulfite and Seawater

Seawater generates particular challenges, considering that its buffer effect does not allow reaching pH 11 with sustainable amounts of reagent [37]. The precipitation of divalent ions forms hydrophilic species that impairs the floatability of valuable minerals such as molybdenite, making it impossible to operate in highly alkaline conditions. In this context, interesting alternatives have been proposed to remove calcium and magnesium ions from seawater that most results in performance drop. For example, Jeldres et al. [155] used a mixture of alkalizing agents (lime, sodium hydroxide, and sodium carbonate), obtaining good recoveries of chalcopyrite and molybdenite with an adequate pyrite depression. Cruz et al. [5] used an atmosphere rich in carbon dioxide to improve the flotation performance of Cu–Mo ore with high clay content. Arias et al. [156] proposed a biotechnological treatment for the removal of divalent ions from seawater using a fluidized bed bioreactor completed with the halotolerant ureolytic strain Bacillus sub-tilis LN8B. However, these treatments still have high costs that prevent their current implementation. That is why the strategy used by industry is to work at natural pH (approximately pH 8) and use depressants for pyrite, such as sodium metabisulfite (MBS).

**Figure 36:**
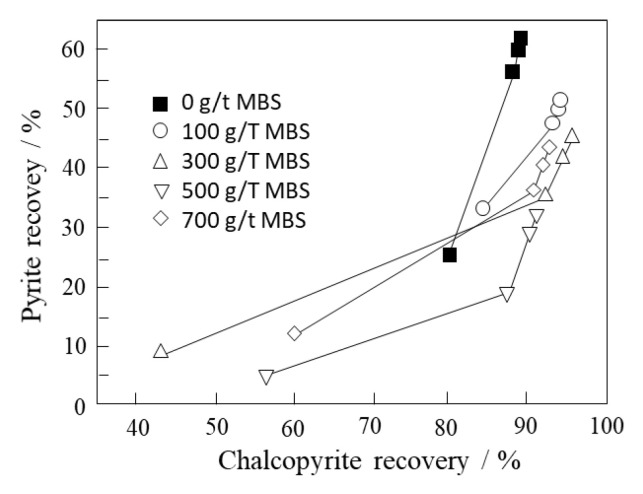
Recovery of pyrite versus chalcopyrite using seawater at pH 8.5. Metabisulfite was added to the mill before grinding. Tests were carried out with artificial seawater and modified thionocarbamate as a collector and MIBC as a frother (adapted from Mu et al. [74]).

An example is shown in Figure 36, obtained from the work of Mu et al. [74]. In the absence of MBS, the recoveries of pyrite and chalcopyrite were high due to the activation of pyrite by Cu^2+^ ions favoring the formation of Cu(I)S species on the surface. As the MBS dosage increases, the recovery of chalcopyrite increases slightly while pyrite decreases.

**Figure 37:**
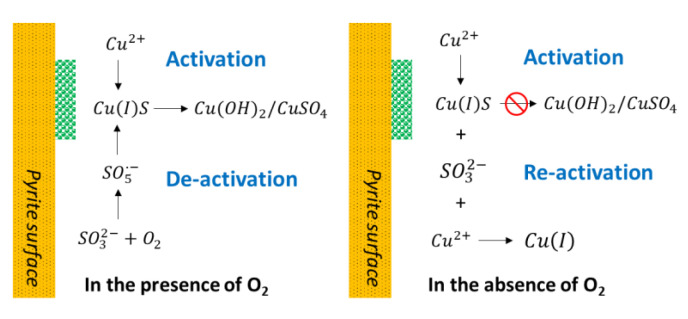
Schematic representation of the effect of MBS on the activation of copper on pyrite in the presence and absence of oxygen (adapted from Mu et al. [74]).

Figure 37 shows a schematic representation of how MBS promotes the formation of hydrophilic species on the pyrite surface, comparing an oxygen-rich system to the case in which oxygen is absent. In the presence of oxygen, the combination of sulfite ions and oxygen can form a strongly oxidizing radical SO_5_^•−^ that can oxidize the Cu(I)S on the surface of pyrite. This forms Cu(OH)_2_/CuSO_4_ of a hydrophilic nature. Then, the more substantial effect of MBS is when it is added in flotation instead of milling since this has a higher oxygen exposure.

Otherwise, when oxygen is absent, MBS cannot depress the flotation of copper-activated pyrite since the formation of SO_5_^•−^ is inhibited, and the sulfite species (${\mathrm{SO}}_{3}^{2-}$) cannot correctly oxidize the Cu(I)S of the surface [74].

### 5.8. Natural Polymers

Several studies have analyzed the effect of natural polymers to depress pyrite, including starch, dextrin, guar gum, and carboxymethylcellulose [157,158,159]. In general, the chemical structure of these reagents is composed of:

(i) a chain of hydrocarbons,
(ii) hydroxyl groups that are distributed throughout the polymer structure, capable of ionizing or forming hydrogen bonds,
(iii) intensely hydrated polar groups such as SO_3_^−2^ and COO^−^, dispersed throughout the molecule.

**Figure 38:**
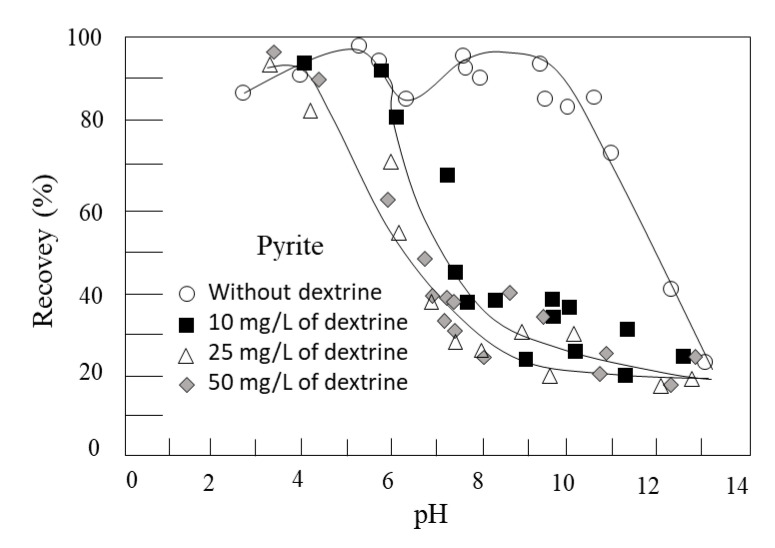
Pyrite floatability with 1 × 10^−4^ mol/L sodium isopropyl xanthate as a function of pH in the absence and presence of various initial concentrations of dextrin [1 g of pure pyrite ore with a nitrogen flow rate of 30 mL/min. Deionized water was used, adjusting the pH with dilute solutions of NaOH and HNO_3_] (adapted from Valdivieso et al. [135]).

**Figure 39:**
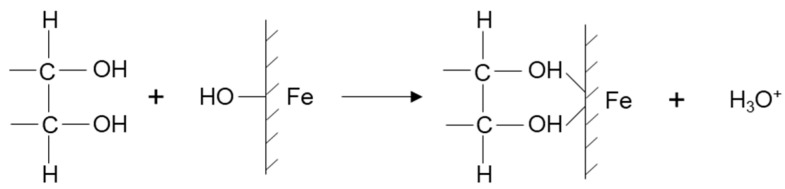
Schematization of dextrin interacting with the ferric oxyhydroxide sites of pyrite (adapted from Valdivieso et al. [135]).

The first systematic work was reported by Valdivieso et al. [135], who showed the effectiveness of using dextrin. Figure 38 shows the floatability of pyrite with isopropyl xanthate in the absence and presence of dextrin as a function of pH. The recovery of pyrite in a polysaccharide-free system was over 80% up to pH 10, to gradually decrease due to the phenomena previously explained. However, as the dextrin concentration increases (10 mg/L approx), the recovery decreases from pH 6. If the dextrin concentration is higher than 25 mg/L, the depression begins at pH 4. At pH < 4, dextrin cannot adhere to the surface, so it does not give it greater hydrophilicity. The authors indicated that the polymer adsorbs on ferric oxyhydroxide sites (Figure 39) while xanthate ions do it on anodic sites located on the non-oxidized surface of pyrite.

**Figure 40:**
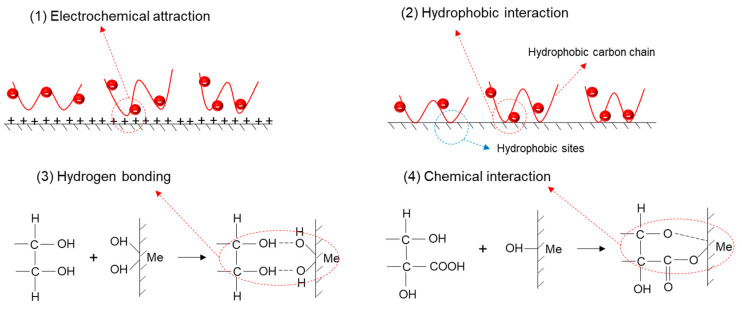
Possible mechanisms of interaction of organic polymers with the mineral surface: (**1**) electrochemical attraction, (**2**) hydrophobic interaction, (**3**) hydrogen bonds, and (**4**) chemical interaction. Figure adapted from (adapted from Mu et al. [160]).

Mu et al. [160] described four mechanisms to explain the adsorption of biopolymers on the mineral surface: (i) electrochemical attraction between oppositely charged polymers with mineral surfaces; (ii) hydrophobic interaction between non-polar segments of polymer chains and the hydrophobic areas of mineral surfaces; (iii) hydrogen bonding formed through the interaction between hydroxyl groups and hydrated metal sites on mineral surfaces, particularly at basic pH; and (iv) chemical interaction between anionic functional groups (such as carboxylate or sulfonic groups) and metal cations on mineral surfaces [161,162] (see Figure 40).

The literature offers many studies that delve into the search for pyrite depressants. Table 5 presents a summary of the main findings reported to date.

## 6. Final Remarks

- Chalcopyrite, the most abundant copper sulfide in nature, is extracted from deposits and concentrated mainly by froth flotation operations. It is common to apply collectors that hydrophobize their surface and generate greater adhesion efficiency to the bubbles to achieve industrially sustainable yields. The most common are xanthates that function efficiently under alkaline conditions and have a high affinity for copper. Xanthate also dissociates to form xanthonic acid ions, thus involving the interaction of copper ions, xanthonic acid, and the surface of chalcopyrite. This phenomenon occurs in two stages: (i) the chemical reaction in solution, where the copper ions react to form the Cu-xanthate complexes; and (ii) the adsorption or chemical reaction on the mineral surface. The copper ions react with the chalcopyrite surface to form CuS, and the xanthonic acid ions are adsorbed onto the Cu. However, screening and/or designing more selective and green collectors is a ‘hot topic’ in the mineral processing field. It is of particular interest to improve yields in copper/pyrite systems, where it is common for a significant portion of the collector to go to iron sulfide. This is caused by the high affinity of the dixanthogen for copper, which usually activates the pyrite surface. The most common strategies include adding lime to bring the pH to highly alkaline conditions, where the dixanthogen is deactivated, and hydrophilic calcium complexes are formed that adhere to the mineral surface.
- It is also possible to work with additional reagents such as pyrite depressants, mainly of an inorganic nature such as metabisulfite or cyanide, with certain recent studies that have promoted the use of natural polymers such as dextrins, guar gum, or carboxymethyl cellulose. Other studies delve into the analysis of more selective reagents, with new families of collectors that could even replace the use of xanthates, considering reagents such as 1-Hydroxyethylidene-1, 1-diphosphonic acid [50], *N*-propyl-*N′*-ethoxycarbonyl thiourea (PECTU) [37], *N*-isopropoxypropyl-*N’*-ethoxycarbonylthiourea [180]. It is noteworthy that recent studies include new generation collectors such as nanocollectors. Although copper minerals have no precedents, promising results have opened a window to delve into this field [181,182,183].
- Fundamental challenges in copper flotation include the processing of minerals with complex gangues such as the presence of clays and the use of low-quality water such as seawater. Neither topic is addressed in detail in this review, considering that there are two recently published publications on this topic. Jeldres et al. [6] analyzed the effect caused by clays, and Cruz et al. [37] delved into the impact of seawater.
- In addition to the froth flotation technique, another technique used to recover chalcopyrite is electroflotation. The basis of this technique is the production of small bubbles (0.008–0.015 mm) through the application of electric current. Electrodes are placed in the mineral pulp, thus causing electrolysis of the water.

Although this technique appeared decades ago, nowadays, it could present a boom mainly due to deeper and deeper deposits where the mineralization is becoming finer. Having liberated mineral species implies an increasing size reduction, where conventional flotation does not respond well. Among the main problems of traditional froth flotation with fine particles are occlusion of the minerals or species of interest and increased entrainment of gangue by the concentrate or valuable species.

Some of the investigations carried out regarding the use of this process applied to chalcopyrite and pyrite minerals showed that in the presence of the xanthate collector, mineral recovery was higher due to the action of oxygen bubbles, created by water electrolysis, where the adsorption rate of xanthate on pyrite and chalcopyrite increases with increasing oxygen concentration [184,185]. Thus, dissolved oxygen’s presence facilitates the collectors’ adsorption on the surface of the minerals. However, a long contact time with oxygen could oxidize and depress the minerals.

## Figures and Tables

**Table 1:** Reagents used in the chalcopyrite flotation.

Reagents	System	Results and Discussion	References
Collector and frother: thiophosphate Depressants: Sodium silicate (SS) and sodium carboxymethyl cellulose (CMC).	Separation of chalcopyrite and galena. Particle size −74 + 38 µm. The pH was 7–7.5. XFG microflotation cell.	Both depressants applied by themselves showed a slight depression of the mineral chalcopyrite and galena. However, the separation was significant using a 200 mg/L 50:1 mixture of SS and CMC, allowing galena to settle without affecting chalcopyrite floatability. An additional dosage of SS and zinc sulfate generated a better separation.	[52]
Collector: potassium butyl xanthate (PBX) Depressant: Calcium lignosulfonate (CLS). Frother: MIBC	Separation of chalcopyrite and talc in deionized water. Particle size −74 + 38 µm. pH = 8 fixed with HCl and NaOH. XFG-C microflotation cell.	With 200 mg/L of CLS, its depressant effect on chalcopyrite increases with the addition of calcium ions (1 × 10^−3^ mol/L). The sulfonic and carboxyl groups of CLS adhere to the surface of pyrite and talc, preventing dixanthogen formation. The flotation separation of chalcopyrite and talc can be achieved at pH 6–12 using the combined depressant of CLS and calcium ions.	[53]
Collector: butyl xanthate and *O*-isopropyl-*N*-ethyl thionocarbamate (IPETC). Depressants: Sodium sulfite and sodium silicate. Frother: oil.	Separation of chalcopyrite and galena in deionized water. Particle size 0.038–0.074 mm. pH = 8.5–9 fixed with HCl and NaOH. XFG microflotation cell.	Chalcopyrite recovery remained above 80% throughout the sodium sulfite dosage range and sodium silicate (0–1000 mg/L). However, the recovery of galena decreased progressively with the increase of the depressant, both sodium sulfite, and silicate, achieving a maximum separation of chalcopyrite and galena at 82.4% and 20.3%, respectively. The Cu^2+^ or Fe^2+^ produced from the dissolution of the chalcopyrite surface did not interact with SO_3_^2−^ while the Pb^2+^, a product of the dissolution of the galena surface, interacted with SO_3_^2−^. This binding resulted in PbSO_3_, which could subsequently oxidize to PbSO_4_ and interact with the water molecules, demonstrating the separation of the two minerals.	[54]
Collector: Potassium ethyl xanthate. Depressant: High molecular weight polyacrylamide (PAM). Frother: n/a	Separation of chalcopyrite and galena in distilled water. Particle size −75 + 38 µm. pH = 9–11 fixed with HCl/NaOH. Nitrogen was used as gas in a microflotation cell.	Chalcopyrite floats at pH = 9–11 with 5 × 10^−4^ mol/L of collector. However, after adding the depressant PAM (8 mg/L), the recovery fell drastically to values close to 5%. In addition, with a 1:1 weight ratio mixture with galena, chalcopyrite reaches up to 30% recovery with 4 mg/L of PAM, and 20% with 8 mg/L. This separation resulted because the PAM-galena bond was weakened after adding KEX, while there was no significant change between PAM-chalcopyrite.	[55]
Collector: *O*-isopropyl-*N*-ethyl thiocarbamate (IPETC). Depressant: Poly (acrylamide-allyl thiourea) (PAM-ATU)/Thioglycolic Acid (TGA). Frother: MIBC.	Separation of chalcopyrite and molybdenite in distilled water. Particle size −74 + 38 µm. pH = 2–11, fixed with HCl and NaOH. XFG5-35 microflotation cell.	With 6 mg/L of collector and 10 mg/L of frother, the recovery of both minerals was 90% (pH 2–11). However, with a dose of 6 mg/L of PAM-ATU, a depression of 75% was observed for chalcopyrite. With 20 mg/L of TGA, the chalcopyrite could be depressed without affecting the molybdenite. At pH = 10.5 with PAM-ATU, the separation between both minerals was effective, being 20% chalcopyrite and 80% molybdenite. Therefore, PAM-ATU exhibits a stronger depressant effect on chalcopyrite than TGA at low doses.	[56]
Collector: sodium butyl xanthate (SBX). Depressant: Xanthan gum (xanthan gum). Frother: MIBC.	Separation of talc and chalcopyrite. Particle size −74 + 38 µm. pH = 2–12 fixed with HCl/NaOH. XFGC microflotation cell.	The flotation of chalcopyrite and talc remained above 90% (pH = 2–12), but adding xanthan gum, the chalcopyrite depressed in acid conditions, recovering 10% at pH = 2. This effect disappears at pH = 8, returning to 90% recovery, even with xanthan gum. The talc depressed highly at pH = 2–9, to later increase its recovery, reaching 80% at pH = 12. In this context, it is inferred that xanthan can achieve selective flotation of the chalcopyrite/talc system at pH = 8.	[57]
Collector: Sodium butyl xanthate. Depressant: Carrageenan (carrageenan). Frother: MIBC.	Separation of talc and chalcopyrite in deionized water. Particle size −74 + 38 µm. pH = 2–12 fixed with HCl and NaOH. XFG microflotation cell.	After adding carrageenan, chalcopyrite did not present changes in its recovery (pH 8), unlike talc. With doses from 0 to 2400 g/t, talc recovery decreased from 97% to 8%. However, raising the pH from 8 to 12, the chalcopyrite recovery drastically reduced from 92% to 22% due to the oxidation of the mineral surface, generating hydrophilic hydroxides.	[58]
Collector: potassium isobutyl xanthate. Depressant: Humic acid (HA). Frother: MIBC	Separation of high purity chalcopyrite and molybdenite in 0.001 M KCl solution. Particle size −150 + 74 µm. pH = 3–11 fixed with KOH and HCl. Hallimond tube microcell.	Unlike chalcopyrite, the recovery of molybdenite was affected by HA (20 ppm), which dropped sharply from 98% to 14%. At 40 ppm, values lower than 10% were obtained. Therefore, a good separation range of pH 3–11 was observed, in which molybdenite is effectively depressed with 20 ppm of HA, keeping the chalcopyrite flotation unchanged. This reduction in molybdenite floatability suggests that chemical interaction is absent between HA and the basal planes of molybdenite, unlike between HA and chalcopyrite, whose interaction is electrostatic.	[59]
Collector: potassium ethyl xanthate (KEX). Depressant: Chitosan Frother: n/a	Separation of chalcopyrite and galena in distilled water. Particle size −75 + 38 µm. pH = 3–9 fixed with HCl and NaOH. Microflotation cell.	With chitosan, both chalcopyrite and galena were significantly depressed. At pH = 3, chalcopyrite recovery reduced from 90% to 60% (with 0.67 mg/L chitosan). In the pH range 3–5, the recovery dropped to 20% and remained so at higher pH. Galena also suffered low recoveries, falling from 91% to 40%. This could suggest that chitosan would not be a selective depressant in Cu–Pb separation. However, by mixing these minerals, competitive adsorption of chitosan in the sulfide occurs, where chalcopyrite has higher absorption than galena.	[60]
Collector: kerosene. Depressant: cupric chloride and sodium sulfide. Frother: terpenic oil.	Separation of chalcopyrite and molybdenite in distilled water. Particle size −100 + 43 µm. pH = 8 fixed with NaOH and HCl. XFG microflotation cell.	Chalcopyrite recovery decreased drastically with increasing concentration of S^−2^, having a recovery of 26% (S^−2^ 50 mg/L). However, there was no significant effect on molybdenite, which could confirm a selective separation of these minerals. The presence of Cu^+2^ was detrimental to the flotation of chalcopyrite and molybdenite. The authors indicated that copper ions drastically reduced the recoveries of copper and molybdenum and increased the inhibition of sodium sulfide; that is, copper ions seriously hampered the flotation separation of chalcopyrite and molybdenite.	[61]
Collector: kerosene. Depressant: L-cysteine. Frother: terpinol	Separation of chalcopyrite and molybdenite in deionized water. Particle size −74 + 45 µm. pH = 4–12 fixed with NaOH and HCl. XFG microflotation cell.	Chalcopyrite and molybdenite achieved 90% and 80% recoveries, respectively (pH 4–12). However, in the presence of 4 × 10^−4^ mol/L of L-cysteine, the recovery of chalcopyrite was reduced by 20% at pH = 4, while at pH = 12, it was reduced to 5%. Changes in molybdenite were insignificant. This reduction in flotability is due to the excellent performance of L-cysteine, which can be attributed to its molecular structure having thiol (-SH) and primary amine (-NH_2_) functional groups, which could coordinate with copper ions.	[62]
Collector: sodium isobutyl xanthate (SIBX). Depressant: dithiothreitol (DTT). Frother: MIBC.	Separation of chalcopyrite and molybdenite in ultrapure water. Particle sizes −75 + 38 µm. pH = 6–12 fixed with NaOH and HCl. XFG microflotation cell.	Chalcopyrite and molybdenite had a good recovery (over 90%) at pH 6–12. However, when using DTT as a depressant, the floatability of chalcopyrite decreased drastically, obtaining a recovery of 8% (pH 9–12). In the case of molybdenite, it was not influenced by DTT, confirming that this depressant is an excellent alternative for Cu–Mo separation. The -SH groups within the DTT molecule strongly complexed with the Cu sites, exposing the OH group bonds towards the water, whereas for molybdenite, the non-polar hydrophobic faces were the main cleavage surfaces had no affinity for DTT, resulting in negligible adsorption.	[63]
Collector: kerosene. Depressant: dithiouracil. Frother: MIBC	Separation of chalcopyrite and molybdenite in deionized water. Particle size −74 + 38 µm. pH = 4–11. XFG microflotation cell.	Chalcopyrite had 80% recovery throughout the experimental pH range (pH 4–11); however, when dithiouracil was introduced, the value was reduced to 23% (pH 4). Molybdenite had a negligible decrease in its recovery upon adding the depressant. Analysis showed that dithiouracil would coordinate with Cu ions by generating dithiouracil-Cu complexes.	[64]
Collector: diesel. Depressant: Sodium hydrosulfide (NaHS). Frother: MIBC	Separation of chalcopyrite and molybdenite in deionized water. Particle sizes of P80 = 106 µm and P80 = 53 µm. pH= 7–11 fixed with NaOH. JK 1.5 L flotation cell.	Chalcopyrite showed good floatability in the absence of NaHS, and the recovery reached almost 90%. However, the final recovery decreased to 47% and 70%, with 6 mmol/L and 9 mmol/L of NaSH, respectively. At 12 mmol/L, chalcopyrite was recovered by less than 5%. The molybdenite did not undergo substantial changes. When NaHS is added, the ionized HS- lowers the Eh, producing a reducing environment where the chalcopyrite surface is free of hydrophobic species such as S^0^. Thus, the collector cannot adhere in the presence of this depressant.	[65]
Collector: kerosene. Depressant: rhodanine-3-acetic acid (3-Rd). Frother: MIBC.	Separation of chalcopyrite and molybdenite in deionized water. Particle size −74 + 38 µm. pH = 4–12 fixed with HCl and NaOH. XFG microflotation cell.	Chalcopyrite and molybdenite had good floatability at pH 4–12. However, by adding the depressant, the recovery was reduced to 30% at pH = 4, while molybdenite was slightly affected. This depression is due to the –COO– and –CSS– functional groups that bind to Fe sites on the mineral surface.	[66]
Collector: sodium butyl xanthate (SBX). Depressant: propylene carboxylic acid (PCA). Frother: MIBC.	Separation of chalcopyrite and pyrite in deionized water. Particle size −74 + 38 µm. pH = 4–12 fixed with HCl and NaOH. XFG microflotation cell.	Chalcopyrite and pyrite show recovery of over 90% when working with SBX and MIBC at pH 4–10. An increase in pH to 11.5 allowed lowering the pyrite recovery to 63%. However, when using PCA, pyrite flotation was significantly reduced at pH 4–8, while chalcopyrite was slightly affected, with recoveries over 90%. At pH 8, with 60 mg/L of PCA and 15 mg/L of SBX, the highest separation was obtained, with recoveries of 1.3% for pyrite and 90% for chalcopyrite, generating a good selective separation.	[67]
Collector: sodium butyl xanthate (SBX). Depressant: zinc sulfate. Frother: MIBC	Separation of chalcopyrite and talc in deionized water. Particle size −105 µm. pH = 5–12 fixed with HCl and NaOH. XFGC II microflotation cell.	Increasing the zinc concentration from 0 to 3 × 10^−3^ mol/L, the recovery of talc decreased from 95% to 20% (pH = 9). Chalcopyrite showed a decrease from 98% to 80%. In this context, it is suggested that ZnSO_4_ could be an attractive talc depressant due to the formation of zinc hydroxides that precipitated on the talc surface, whose process was facilitated by Mg^2+^.	[68]
Collector: sodium ethyl xanthate (SEX). Depressant: starch (Starch). Frother: FZS180 (polyglycol ethers).	Separation of chalcopyrite and graphite in tap water. Particle size P80 = 200 µm. pH = 7.5 fixed with HCl and NaOH. 1.5 L mechanical flotation cell.	Chalcopyrite and graphite recoveries were greater than 90% after 10 min of flotation without starch. By adding starch at 8 mg/L, it was observed that the recovery of chalcopyrite was reduced to 82%, while graphite to 78%. With doses of 20 mg/L, 48% and 46% recoveries were obtained. With 33 mg/L, recoveries were around 12% in both minerals, so the starch is not a selective depressant. However, when using oxidized starch, a selective separation could be observed at doses of 5 and 20 mg/L, achieving a separation difference of 37%.	[69]
Collector: n/a. Depressant: n/a. Frother: n/a. Modifier: sodium silicate (Na_2_SiO_3_, SS).	Flotation of chalcopyrite in seawater, pure water, and water with 0.05 M MgCl_2_. Particle size 38–75 µm. pH 10 fixed with NaOH. XFGⅡ5-35 microflotation cell.	In seawater and solutions with 0.05 M MgCl_2_, chalcopyrite showed a significant depression at pH 10 due to the formation of Mg(OH)_2_ precipitates on the mineral surface. However, after adding sodium silicate, the chalcopyrite shows an increase in the recovery by flotation due to the adsorption of the reagent on the Mg(OH)_2_ particles. This behavior was not shown in freshwater, so the flotation of chalcopyrite in seawater is adequate with sodium silicate.	[70]
Collector: kerosene. Depressant: sodium thiosulfate. Frother: MIBC.	Separation of chalcopyrite and molybdenite in deionized water. Particle size −74 + 38 µm. pH = 2–12 fixed with HCl and NaOH. XFG microflotation cell.	Sodium thiosulfate flotation showed that the depression for chalcopyrite is much larger than for molybdenite. However, after adding Cu^2+^ ions (3 × 10^−5^ mol/L), there was an optimal selective separation of these minerals, where the floatability difference reached 74.9 (49.9% in the absence of Cu^2+^). Zeta potential measurements illustrated that sodium thiosulfate and copper ions could be selectively adsorbed on the chalcopyrite surface.	[71]
Collector and frother: dibutyl dithiophosphate. Depressant: sodium alginate.	Separation of chalcopyrite and galena in deionized water. Particle size −106 + 74 µm. pH = 7–11 fixed with HCl and NaOH. XFG microflotation cell.	Without depressant, the recoveries of both minerals were around 90% (pH 7–11), demonstrating the efficient use of the collector but its null selective capacity. However, in the presence of 15 mg/L NaAl (sodium alginate), the recovery of chalcopyrite remained high, but that of galena decreased to less than 20% (pH = 7–12). Even as the concentration of NaAl increases, the recovery of galena decreases, proving the efficient capacity of this depressant to separate both minerals selectively. The authors hypothesized that sodium alginate could selectively adsorb to the galena surface, hindering subsequent adsorption of the dithiophosphate collector.	[72]
Collector: sodium butyl xanthate (SBX). Depressant: tannic acid (TA). Frother: terpenic oil.	Separation of chalcopyrite and pyrite in deionized water. Particle size −74 + 38 µm. pH = 8 fixed with HCl and NaOH. Microflotation cell n/a.	The recovery of chalcopyrite and pyrite was high with low doses of the collector (10 mg/L) at pH = 8. However, this was modified with the addition of TA, where the pyrite recovery decreased from 70% to 7%, with 200 mg/L. The chalcopyrite slightly lowered its floatability. The authors posited that TA is selectively adsorbed on the pyrite surface by interaction with active Fe atoms, forming large hydrophilic groups.	[73]
Collector: thionocarbamate and alkyl mercaptan. Depressant: sodium metabisulfite (MBS). Frother: MIBC.	Separation of chalcopyrite and pyrite in synthetic seawater. Particle size D80 of 106 µm. pH 8.5 fixed with lime and HCl. 1.5 L Agitair flotation cell.	The addition of 300 g/t of MBS recovered 95% chalcopyrite and 30% pyrite. With 700 g/t, the pyrite recovery was 10% higher, reaching 40%, without changing the recovery of chalcopyrite. According to the results, MBS can depress copper-activated pyrite, depending on the presence of oxygen. In the absence of oxygen, MBS can only promote copper activation on the pyrite surface if copper ions are present. Oxygen allows the formation of copper hydroxides on the pyrite surface, improving the depressant effect.	[74]
Collector: diphosphonic acid (HEDP) and ethyl xanthate (EX). Depressant: n/a Frother: n/a	Separation of chalcopyrite and pyrite in deionized water. Particle size −74 + 35 µm. pH = 3–11 fixed with HCl and NaOH. XFG microflotation cell.	With 2 × 10^−4^ mol/L of HEDP, there was a slightly higher recovery compared to EX. However, the results were reversed for pyrite. It was shown that HEDP could achieve the selective separation of chalcopyrite from pyrite at pH 9, unlike the traditionally used EX.	[50]
Collector: sodium butyl xanthate (NaBX). Depressant: pyrogallic acid (PA). Frother: terpenic oil.	Separation of pyrite and chalcopyrite in deionized water. Particle size −74 + 38 µm. pH = 8 fixed with HCl and NaOH. XFG-II microflotation cell.	The authors analyzed the effect of the depressant PA. When the reagent was not added, pyrite recovery was 70% and chalcopyrite 90% (pH = 8). Adding depressant, with a concentration of PA in the pulp of 200 mg/L, the recovery of pyrite was 3%, while that of chalcopyrite remained above 90%. This shows that PA is a selective pyrite depressant. The analyses showed that the binding of NaBX on the pyrite surface is hindered due to selective adsorption of PA, producing a passivation layer. This selectively depresses pyrite flotation.	[75]
Collector: n/a. Depressant: CaCl2–MgCl2. Frother: n/a	Separation of chalcopyrite (75–106 µm) and molybdenite (<38 µm). The water contained Ca^2+^ ions (0–1 × 10^−2^ M) and Mg^2+^ ions (0–1 × 10^−2^ M). pH = 4–11 fixed with HCl and KOH. Hallimond tube microflotation.	Ca^2+^ and Mg^2+^ ions depress the natural floatability of molybdenite and chalcopyrite minerals at pH > 9. The authors justified the results by forming Mg(OH)_2_ and CaCO_3_ precipitates, which are deposited on the surface of the minerals.	[76]
Collector: butyl xanthate (BX) and pine oil. Depressant: mercaptoacetic acid. Frother: pine oil.	Separation of chalcopyrite and galena mineral in deionized water. Particle size −74 + 38 µm. pH = 2–12 fixed with HCl and NaOH. XFGⅡ5-35 microflotation cell.	At pH = 6 and 0.04 mol/L of BX, chalcopyrite recovery decreased from 83% to 34% after adding mercaptoacetic acid. Galena suffered an insignificant decrease. With 3-mercaptopropionic acid, the recovery of chalcopyrite had a drastic reduction of up to 3%, while galena had a gradual decline. In the case of 3-mercaptoisobutyric acid, the recovery of chalcopyrite reached up to 10%. 3-mercaptopropionic acid is the reagent that shows the strongest depression among the three depressants.	[77]
Collector: sodium butyl xanthate. Depressant: polyglutamic acid (PGA). Frother: terpenic oil.	Separation of chalcopyrite and pyrrhotite. Particle size −74 + 38 µm. pH = 8 fixed with NaOH and HNO_3_. XFG microflotation cell.	The addition of 20 mg/L PGA allowed a selective separation of chalcopyrite in the presence of pyrrhotite, where a higher selectivity towards iron-containing sulfide bands was found in Cu–Fe flotation systems. Surface measurement techniques showed that PGA adsorbed and significantly modified the surface properties of pyrrhotite, improving its hydrophilic character.	[78]

**Table 2:** Stability pH of hydrogen sulfide collectors.

Collector	pH Range
Xanthate	8–13
Dixanthogen	1–11
Dithiophosphate	4–12
Dithiocarbamate	5–12
Thionocarbamate	4–9
Mercaptobenzothiazole	4–9

**Table 3:** Critical pH values of different sulfide minerals (ethyl xanthate collector).

Mineral	Critical pH
Pyrrhotite	6.0
Arsenopyrite	8.4
Galena	10.4
Pyrite	10.5
Marcasite	11.0
Chalcopyrite	11.8
Covellite	13.2
Bornite	13.8
Chalcocite	>14

**Table 4:** Resting potentials of sulfide minerals and interaction product between these minerals in the presence of 6.25 × 10^−4^, pH 7, ethyl xanthate [113].

Mineral	Rest Potential, V	Surface Product
Pyrite (FeS_2_)	0.22	Dixanthogen
Arsenopyrite (FeAsS)	0.22	Dixanthogen
Pyrrhotite (Fe_1−x_S)	0.21	Dixanthogen
Molybdenite (MoS_2_)	0.16	Dixanthogen
Chalcopyrite (CuFeS_2_)	0.14	Dixanthogen
Covellite (CuS)	0.05	Dixanthogen
Bornite (Cu_5_FeS_4_)	0.06	Metallic xanthogen
Galena (PbS)	0.06	Metallic xanthogen

**Table 5:** Summary of studies of pyrite depressants in flotation stages.

Reagent	System	Results and Discussion	References
Collector: potassium ethyl (PEX) and sodium propyl (SPX). Depressant: dextrin. Frother: n/a	Pure pyrite. Particle size −75 + 45 µm. pH = 8 fixed with HCl/KOH. Hallimond microflotation cell.	With 1 × 10^−3^ mol/dm^3^ of ethyl and propyl xanthate, the pyrite recovered close to 80%. However, after adding dextrin (50 mg/L), the recovery reduced to 10%. This was achieved when the pyrite was oxidized for 30 min, while with oxidation of 24 h, the recovery reached 20%. Pyrite depression is given by the formation of dextrin bonds with the Fe–OH groups of the ferric hydroxide formed, thus converting the positive zeta potential of pyrite to negative.	[163]
Collector: sodium isopropyl xanthate. Depressant: Dextrin Frother: n/a.	Pyrite in deionized water with 0.01 mol/L of NaNO_3_. Particle size −150 + 70 µm. pH = 2−12 fixed with HNO_3_/NaOH. Hallimond microflotation cell.	Dextrin adsorption at pH < 4 was insignificant due to the production of ferric hydrosulfate as the main oxidation product on the mineral surface. However, at higher pH, dextrin adsorption is enhanced by the appearance of ferric oxyhydroxide. With 10 mg/L of dextrin, the recovery was 30% at pH 8 and 20% at pH = 12.	[135]
Collector: Potassium ethyl xanthate (KEX). Depressant: Chitosan. Frother: n/a.	Pyrite and galena in distilled water. Particle size −75 + 38 µm. pH = 3−9 fixed with HCl/NaOH. Flotation cell: n/a	Pyrite and galena flotation with KEX had recoveries greater than 90% for each mineral (pH = 8). After adding chitosan (0.67 mg/L), the pyrite recovery decreased to 23% (pH 4−9). In a flotation with mixtures of pyrite and galena, the recovery of galena was much higher than separately, concluding that chitosan is preferentially adsorbed on pyrite.	[60]
Collector: ethyl xanthate. Depressants: quebracho (tannin). Frother: n/a.	Pyrite and chalcopyrite in distilled water. Particle size −150 + 45 µm. pH = 4–10 fixed with HCl/NaOH. Hallimond microflotation cell.	Pyrite recovery decreased from 90% to 48% after adding tannin (0.25 g/L, pH = 8). Without tannin, the depressant effect began at pH 7, but after adding the depressant, the recovery was reduced throughout the pH range, showing a more intense effect in the alkaline range (pH = 8–10). The depressant action of quebracho is a function of the content of the –OH group.	[164]
Collector: potassium amyl xanthate (PAX). Depressant: lignosulfonate biopolymers (DP-1775, DP-1777, and DP-1778). Frother: NASCOL 442.	Pyrite in deionized water. Particle size P80 = 106 µm. pH = 5 fixed with H_2_SO_4_/NaOH. 1.5 L JK flotation cell.	Without activation by copper, pyrite lowered its recovery with the three hydrophilic biopolymers. When the biopolymer concentration was 7 mg/L, DP-1775 and DP-1778 slightly depressed pyrite, but DP-1777 did not show depression. When the biopolymer concentration increased to 33 mg/L, all three biopolymers depressed pyrite. The recovery reduction was strongest at this concentration with DP-1778 (16%), followed by DP-1775 and DP-1777 (21% and 32%, respectively). The molecular weight causes the depressive effect; the higher the molecular weight, the higher the adsorption capacity, and the greater the coverage of the biopolymers on the pyrite surface.	[108]
Collector: potassium isobutyl xanthate (KIBX). Depressant: PAM with different functional groups, hydroxyl (PAM-H), thiourea (PAM-T), carboxyl (PAM-C), and sulfonate (PAM-S). Frother: aerofroth 65.	Sphalerite and pyrite in demineralized water. Particle size −3.2 + 0.6 mm. pH = 11.5 fixed with NaOH. 1.5 L Agitair flotation cell.	The mineral separation increased with PAM, with a more significant depression of pyrite than sphalerite. With PAM-C and PAM-S (500 g/t), pyrite recovery decreased by 25% and sphalerite by 10%. PAM-C is a more selective depressant than PAM-S. Likewise, the PAM-H and PAM-T depressants were stronger but less selective. As expected, better depression of pyrite and better mineral separation was obtained when the polymers were added before the collector.	[161]
Collector: butyl xanthate. Depressant: sodium glycerine-xanthate (SGX). Frother: MIBC. Modifier: Cu^2+^ as activator.	Pyrite and marmatite. Particle size: no. pH = 4–12 fixed with HCl/NaOH. 40 mL microflotation cell.	The recovery of pyrite and marmatite was reduced under alkaline conditions. At pH = 9, the recovery was 55%. In the presence of cupric ions (1 × 10^−4^ mol/L), the recovery of both marmatite and pyrite increased at pH < 11. However, the floatability of pyrite decreases after the application of SGX, being completely inhibited at pH > 10. The –OH and –CSS bonds of the SGX molecule compete with the collector for the mineral surface. The hydrophilic groups are adsorbed on the surface, thus inhibiting its floatability.	[165]
Collector: butyl xanthate (KBX). Depressant: sodium hypochlorite (Ca(ClO)_2_). Frother: pine oil.	Pyrite in deionized water. Particle size −74 + 38 µm. pH = 5–13 fixed with H_2_SO_4_/NaOH. 40 mL RK-FGC5 microflotation cell.	In the absence of Ca(ClO)_2_, and with 1 × 10^−3^ mol/L of collector, the pyrite had good floatability (78–90%) at pH = 5–11. After adding 100 mg/L of depressant, and as the pH increased, the pyrite was depressed, falling from 52% to 31% from pH 6 to pH 13. The addition of Ca(ClO)_2_ forms hydrophilic species on pyrite surfaces and repels dixanthogen adsorption.	[166]
Collector: sodium butyl xanthate (SBX). Depressant: sodium tricarboxylate starch (TCSS). Frother: terpineol.	Pyrite and chalcopyrite in deionized water. Particle size −74 + 38 µm. pH = 2–12 fixed with NaOH/HCl. XFG microflotation cell (40 mL).	The SBX collector displayed an excellent selectivity in the Cpy-Py separation. An analysis of pure minerals showed that TCSS depressed pyrite, lowering its floatability from 90% to 20%. The TCSS adsorption was through a chemisorption mechanism, which passivated the surface and inhibited adsorption and subsequent oxidation of the collector.	[22]
Collector: sodium butyl xanthate (SBX). Depressant: locust bean gum (LBG). Frother: terpenic oil.	Pyrite and chalcopyrite in deionized water. Particle size of −74 + 38 µm. pH = 8 fixed with HCl/NaOH. Microflotation cell (40 mL).	With the addition of the SBX collector (5–200 mg/L), the recoveries of pyrite and chalcopyrite were 92 and 97%, respectively. However, after adding LBG (50 mg/L), pyrite recovery dropped to 10%, slightly affecting chalcopyrite. The authors proposed that the adsorption of LBG on the pyrite surfaces was due to acid/base interactions and the formation of hydrogen bonds between the hydroxylated surfaces of the pyrite and the hydrophilic OH^−^ groups of pyrite and the single bonds of LBG molecules.	[167]
Collector: sodium isopropyl xanthate (SIPX). Depressant: zinc sulfate. Frother: polypropylene oxide methanol.	Pyrite and chalcopyrite. Particle size d90 = 45 µm. pH = 9 fixed with Na_2_CO_3_. 1.5 L Agitair flotation cell.	Zinc sulfate was added to the pulp conditioned at Eh = 275 mV (SHE). In 8 min, pyrite recovery decreased from 58% to 34% with zinc sulfate (2000 g/t), while chalcopyrite increased from 76% to 81%. The mineral depression was attributed to the adsorption/precipitation of zinc hydroxide on the surface under slightly alkaline pH by electrostatic interaction with the ferric hydroxide groups.	[125]
Collector: aerophine 3418 A. Depressant: sodium metabisulfite (Na_2_S_2_O_5_) and starch (starch). Frother: MIBC.	Pure pyrite. Particle size −100 µm. pH = 6.5 and 10 fixed with lime and H_2_SO_4_. 1 L flotation cell.	A dosage of 40 mg/L of collector and 10 mg/L of frother was applied. After adding starch (17 mg/L), the pyrite recovery decreased from 44% to 10% at pH 10 and from 75% to 59% at pH = 6.5. In the case of sodium metabisulfite, the pyrite recovery decreased from 43% to 4% at pH = 10 and from 75% to 23% at pH= 6.5 with a depressant dosage= 134 mg/L. This reduction in floatability is due to the sulfite ions, which have a higher affinity for the surface sites, unlike the adsorbed species of the collector.	[168]
Collector: potassium ethyl xanthate (KEX). Depressant: sodium sulfite.	Pyrite and sphalerite in ultrapure water (0.001 mol/L KNO_3_). Particle size 20 µm. pH = 8.5 fixed with HNO_3_/KOH. Smith & Partridge microflotation cell.	Adding 2 × 10^−4^ mol/L of sodium sulfite, the depression of pyrite was pronounced, lowering its recovery from 37% to 13%. This was caused by the oxidation of copper on the surface of the ore, generating copper hydroxide/oxide that inhibits the adsorption of the collector.	[146]
Collector: sodium isobutyl xanthate (SIBX). Depressant: sodium bisulfite (NaHSO_3_). Frother: polypropylene oxide methanol.	Pyrite in deionized water. Particle size d80 = 38 µm. pH = 7 and 9 fixed with carbonate/bicarbonate and HCl. Gliwice 500 mL flotation cell.	Pyrite flotation was induced by xanthate (1.1 × 10^−4^ mol/L) and activated by copper cations (2.6 × 10^−4^ mol/L). In the presence of sulfite (1.9 × 10^−3^ mol/L), the recovery dropped from 70% to 53% (pH 7) and from 69% to 58% (pH 9). Pyrite depression by the interaction of sulfite with isobutyl xanthate in solution and lower xanthate adsorption to the mineral surface due to the lack of oxygen in the solution, thus limiting the formation of dixanthogens.	[150]
Collector: potassium amyl xanthate (PAX) and sodium diisobutyl dithiophosate (DTP). Depressant: n/a. Modifier: hydrogen peroxide (H_2_O_2_) at 30% as oxidant.	Pyrite and arsenopyrite in demineralized water. Particle size −75 + 38 µm. pH = 6.4 fixed with H_2_SO_4_/NaOH. IMN flotation cell.	Applying 120 g/t of DTP, recoveries of 78% and 36% of pyrite and arsenopyrite were obtained. With PAX, pyrite recovery was the same as DTP, while arsenopyrite had a 33% recovery. After the addition of hydrogen peroxide (2 kg/t) and PAX (120 g/t), the best separation efficiency was obtained, with recoveries of 63% (pyrite) and 5% (arsenopyrite). Oxidation of adsorbed xanthate ions on hydrophilic monothiocarbonate ions was the most apparent reason for the notable depression of arsenopyrite compared to pyrite.	[169]
Collector: butyl xanthate. Depressant: sodium dimethyl dithiocarbamate (SDD). Frother: pine oil.	Pyrite and chalcopyrite in deionized water. Particle size −74 + 45 µm. pH = 5–11 fixed with HCl and NaOH. XFG flotation cell.	Pyrite recovery at pH 8.5 was 80%, while at pH 11, it dropped to 27%. Adding SDD (2.5 × 10^−4^ M), pyrite recovery decreased at both pHs, dropping to 24% at pH 8.5. The pH had little influence on the flotation of chalcopyrite. SDD selectively adsorbed on the pyrite surface and reduced its hydrophobicity.	[170]
Collector: Sodium Butyl Xanthate (SBX). Depressant: salicylic acid (SA). Frother: terpenic oil.	Pyrite and chalcopyrite in deionized water. Particle size −74 + 38 µm. pH = 8 fixed with HCl and NaOH. 40 mL flotation cell.	Chalcopyrite and pyrite recoveries were 95% and 70%, respectively (10 mg/L of collector). Then, with the addition of SA (200 mg/L), pyrite recovery decreased significantly to 3%, while chalcopyrite did not show significant changes. The authors proposed that SA could be adsorbed on the pyrite surface by interaction with active Fe atoms, forming hydrophilic groups.	[171]
Collector: isopropyl xanthate. Depressant: acidithiobacillus ferrooxidans bacteria. Frother: n/a.	Pyrite in artificial seawater. Particle size P80 = 242 µm. pH = 4 and 8. Flotation cell: n/a.	The results showed a biosuppression of pyrite, increasing the pH from 4 to 8 and lowering the recovery from 92% to 36%. This result was accompanied by increased bacterial adhesion density, from 2.58 × 10^8^ bacteria to 1.99 × 10^9^ bacteria, at pH 4 and 8, respectively. According to this, it can be inferred that the higher the binding density of the bacteria, the greater the depressant effect.	[172]
Collector: potassium butyl xanthate (PBX). Depressant: Konjac glucomannan. Frother: MIBC.	Pyrite and chalcopyrite in distilled water. Particle size −74 + 38 µm. pH = 3–11 fixed with HCl/NaOH. XFG-C flotation cell.	Chalcopyrite and pyrite flotation was higher than 90% (pH 3–10), but after adding konjac glucomannan (10 mg/L), pyrite recovery was less than 5% when the pH changed from 5 to 11. In contrast, the recovery of chalcopyrite remained above 90% throughout the pH range. This results from hydrogen bonds between the oxidized product Fe(OH)_3_ on the pyrite surface and the depressant OH formed through the Bronsted acid-base interaction.	[173]
Collector: sodium isobutyl xanthate (SIBX). Depressant: glucan. Frother: diethylene glycol dimethyl ether (DIDE).	Pyrite and pyrophyllite in distilled water. Particle size −74 + 38 µm. pH = 3–11 fixed with HCl/NaOH. XFG microflotation cell (40 mL).	A pyrite concentrate with a grade of 75.2% and recovery of 95.4% was achieved with 400 g/t of glucan (natural pH). The application of glucan significantly improved the metallurgical performance compared with conventional depressants. The FTIR, XPS, and MDS analysis demonstrated that the non-ionic glucan could interact more intensely on the pyrophyllite surface than on the pyrite surface. The glucan adsorption (chemisorption and physical interaction) on pyrophyllite occurred between the Al/Si atoms on the pyrophyllite surface and the eOH hydrophilic groups in the glucan molecule. The AleO chemical complexation dominated the interaction.	[174]
Collector: potassium butyl xanthate (PBX). Depressant: serpentine mineral (lizardite) (−10 µm). Frother: MIBC.	Pyrite and galena in deionized water. Particle sizes −150 + 74 µm, −74 + 37 µm and 10 µm. pH fixed with HCl and NaOH. FGC5-35 flotation cell.	Pyrite and galena recoveries increased rapidly with increasing PBX concentration, reaching their maximum at 0.5 × 10^−4^ mol/L. Galena was not sensitive to the particle size, achieving recoveries above 80% in all cases. However, the floatability of pyrite was affected by reducing the size: below 10 µm, there were recoveries of 60%, but above 74 µm, the recovery was 95%. After adding 1 g/L of serpentine at pH = 7, pyrite was depressed in all sizes, while galena flotation was slightly affected.	[175]
Collector: ammonium dibutyldithiophosphate (ADD). Depressant: calcium hypochlorite (Ca(ClO)_2_). Frother: n/a.	Pyrite and covellite in deionized water. Particle size −106 + 45 µm. pH = 4–11 fixed with HCl/NaOH. XFG flotation cell.	At 40 mg/L of ADD, the recoveries were 88% for covellite and 77% for pyrite. Pyrite and covellite had high floatability in a range of pH = 4–8, but covellite increased at pH > 8. After adding the depressant (200 mg/L), it was observed that Ca(ClO)_2_ had little effect on covellite flotation. However, pyrite flotation was severely depressed, dropping sharply to 12%. The depression of the pyrite mineral was given by oxidation of the pyrite surface producing Fe(OH)_3_ and SO_4_^2−^. These products formed prevented the adsorption of the collector on the pyrite.	[176]
Collector: isopropyl ethylthiocarbamate (F1234). Depressant: native wheat starch (NWS), wheat starch oxidized with hydrogen peroxide (Perox 3/30), and carboxymethylcellulose (CMC). Frother: polyglycol ethers (polyfroth W34).	Pure pyrite in distilled and deionized water. Particle size P80 = 106 µm. pH = 9 fixed with NaOH/HCl. 1.5 L flotation cell.	Pyrite recovery without copper activation was 33%, and activated pyrite was 76%. Increasing the NWS addition from 100 to 700 g/t, the recovery decreased from 67% to 4%. With Perox 3/30, the depression was more marked than NWS at doses from 100 g/t to 300 g/t. CMC’s recovery is little changed from 21% at 300 g/t to 16% at 700 g/t. These depressants can form a layer on the pyrite surface that prevents interaction with the collector. The starches, NWS, and Perox 3/30 can associate more hydroxyls per adsorption site CMC.	[177]
Collector: potassium isobutyl xanthate (SIPX). Depressants: guar gum with high molecular weight (HMW), namely KU9, guar gum with low molecular weight (LMW), namely CZD535, CMC with a high degree of substitution (CMC HDS), namely Dep386, and low degree of substitution (CMC LDS) namely Dep347. Frother: n/a.	Pure pyrite in distilled water. Particle size −150 + 75 µm. pH = 5–11 fixed with NaOH/HCl. UCT flotation cell.	The maximum recoveries (80% approximately) were obtained at pH 5 and pH 9 with 10^−4^ M of SIPX. However, with 10 ppm of guar gum, the depression was more significant, reaching a recovery close to 5% at pH = 9. CMC can depress pyrite, but at high doses (500 ppm), achieving a recovery of 20% at pH = 9. 200 ppm of CMC at pH 10 caused 10% of the recovery. Therefore, guar gums are stronger depressants than CMCs and are effective at low doses. CMC does not adsorb efficiently on pyrite because of electrostatic repulsion between highly charged substituted groups and the anionic surface of pyrite.	[136]
Collector: butyl xanthate. Depressant: polyacrylamide (PAM) and hydroximic polyacrylamide (HPAM). Frother: n/a.	Pyrite ore in pure water. Particle size −74 + 37 µm. pH = 3–11 fixed with NaOH/HCl. Microflotation cell.	Pyrite recovery was 90% at pH 3–7 and 80% at pH 8–11. However, with 40 mg/L of PAM, the recovery reduced to 60% at pH = 11. HPAM (40 mg/L) caused a dramatically reduction to 50% at pH = 4 and 10% at pH = 11. This can be explained by the high dissociation of HPAM in alkaline solutions leading to an intense interaction between HPAM and the mineral surface.	[178]
Collector: PAX. Depressant: guar gum. Frother: MIBC.	Pure pyrite in seawater. Particle size −65 + 38 µm. pH = 8 (natural). Partridge-Smith microflotation cell.	A maximum recovery of 80% pyrite was observed with 75 ppm PAX and 20 ppm MIBC. However, the recovery decreased to 20% with 75–100 ppm guar gum after adding guar gum. Higher doses had no depressant effect. The depression of the pyrite mineral is given by the action of the guar gum, which adheres to the Fe(OH)_3_ sites formed and thus competes with the bonds of the PAX collector on the surface.	[179]

## Data Availability

The data presented in this study are available on request from authors C.I. Castellón and R.I. Jeldres.
